# Functions of SRPK, CLK and DYRK kinases in stem cells, development, and human developmental disorders

**DOI:** 10.1002/1873-3468.14723

**Published:** 2023-09-04

**Authors:** Elizabeth K. J. Hogg, Greg M. Findlay

**Affiliations:** ^1^ The MRC Protein Phosphorylation and Ubiquitylation Unit, School of Life Sciences University of Dundee UK

**Keywords:** CLK, differentiation, DYRK, embryonic development, HIPK, intellectual disability, kinase signalling, neurodevelopment, phosphorylation, SRPK, stem cells

## Abstract

Human developmental disorders encompass a wide range of debilitating physical conditions and intellectual disabilities. Perturbation of protein kinase signalling underlies the development of some of these disorders. For example, disrupted SRPK signalling is associated with intellectual disabilities, and the gene dosage of DYRKs can dictate the pathology of disorders including Down's syndrome. Here, we review the emerging roles of the CMGC kinase families SRPK, CLK, DYRK, and sub‐family HIPK during embryonic development and in developmental disorders. In particular, SRPK, CLK, and DYRK kinase families have key roles in developmental signalling and stem cell regulation, and can co‐ordinate neuronal development and function. Genetic studies in model organisms reveal critical phenotypes including embryonic lethality, sterility, musculoskeletal errors, and most notably, altered neurological behaviours arising from defects of the neuroectoderm and altered neuronal signalling. Further unpicking the mechanisms of specific kinases using human stem cell models of neuronal differentiation and function will improve our understanding of human developmental disorders and may provide avenues for therapeutic strategies.

## Graphical abstract

This review highlights the established and emerging roles of the SRPK, CLK, and DYRK kinase families in development. Kinase functions elucidated in biochemical and genetic studies inform the underlying mechanisms of human development, whereby kinase‐associated phenotypes arising from developmental models can parallel features of human development disorders.

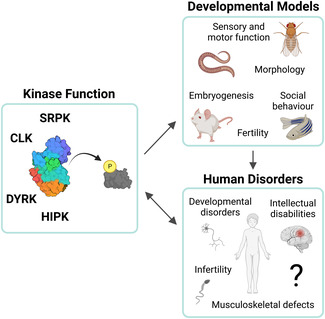

## Abbreviations

**AKT**, AKT/PKB/RAC serine/threonine‐protein kinase

**BMP**, bone morphogenetic protein

**CDC25A**, M‐phase inducer phosphatase

**CDK**, cyclin‐dependent kinase

**c‐JUN**, transcription factor Jun

**CLK**, cdc2‐like kinases

**CMGC**, CDK, MAPK, GSK, and CDK‐like kinase family

**c‐MYC**, myc proto‐oncogene protein

**CPSF**, cleavage and polyadenylation specificity factors

**CREB**, cAMP‐responsive element‐binding protein

**CRMP**, collapsin response mediator protein

**CtBP**, C‐terminal binding protein

**DCAF7**, DNA binding protein 1 and CUL4‐associated factor 7

**DH domain**, DYRK homology box

**Doa**, darkener of apricot (*Drosophila* CLK)

**DREAM**, dimerization partner, retinoblastoma‐associated protein‐like, E2F and multi‐vulval class B complex

**DYRK**, dual‐specificity tyrosine‐regulated kinase

**E14.5**, embryonic day 14.5 (etc.)

**EDVP**, E3 ubiquitin‐protein ligase UBR5, DNA binding protein 1, and Viral protein R binding protein E3 ligase complex

**EGF**, epidermal growth factor

**ERK**, extracellular signal‐regulated kinase

**ESEs**, exonic splicing enhancers

**FGF**, fibroblast growth factor

**GABA**, gamma‐aminobutyric acid

**GLI1**, zinc finger protein GLI1

**GSK3β**, glycogen synthase kinase‐3 beta

**HDAC**, histone deacetylase

**hESC**, human embryonic stem cell

**Hh**, Hedgehog

**HID**, homeobox‐interacting domain

**HIP1**, huntingtin‐interacting protein

**HIPK**, homeodomain‐interacting protein kinase

**hiPSC**, human induced pluripotent stem cell

**HSF**, heat shock factor

**Lrp4**, low‐density lipoprotein receptor‐related protein 4

**MAPK**, mitogen‐activated protein kinase

**MBK**, minibrain kinase

**MEF2C**, myocyte enhancer factor 2

**MNB**, minibrain

**mTORC1**, serine/threonine‐protein kinase mammalian target of rapamycin complex 1

**NAPA**, N‐terminal auto‐phosphorylation accessory region

**NDEL1**, nuclear distribution element‐like 1

**NFAT**, nuclear factor of activated T‐cells

**NLS**, nuclear localisation signal

**O‐GlcNAc**, O‐linked β‐*N*‐acetylglucosamine

**OMA‐1**, CCCH‐type zinc finger protein oma‐1

**p53**, cellular tumour antigen p53

**PAX6**, paired box protein Pax‐6

**PEST**, proline, glutamic acid, serine and threonine rich sequence

**PHLPP1**, PH domain leucine‐rich repeat‐containing protein phosphatase 1

**PIN1**, peptidyl‐prolyl cis‐trans isomerase

**Plk1**, Polo‐like kinase 1

**PRAS40**, proline‐rich AKT substrate of 40 kDa

**PTCH**, protein patched homologue

**REST**, RE1‐silencing transcription factor

**RNF12**, E3 ubiquitin‐protein ligase RLIM

**RS**, arginine/serine sequences

**scRNAseq**, single‐cell RNA sequencing

**SIAH‐1/2**, E3 ubiquitin‐protein ligase SIAH1/2

**SMAD**, mothers against decapentaplegic homologue

**snRNP**, small nuclear ribonucleoprotein

**SR**, serine/arginine sequences

**SRPK**, SR protein kinase

**SRSF**, SR‐rich splicing factor

**SUMO‐1**, small ubiquitin‐related modifier 1

**TGFβ**, transforming growth factor beta

**TOKAS**, Tonne‐Kalscheuer syndrome

**VEGF**, vascular endothelial growth factor

**WNT**, derived from segment polarity gene *wingless* in *Drosophila* and the proto‐oncogene *int‐1*

**YAP**, Yes‐associated protein

**ZFP42/REX1**, zinc finger protein 42 homologue

**Fig. 1:**
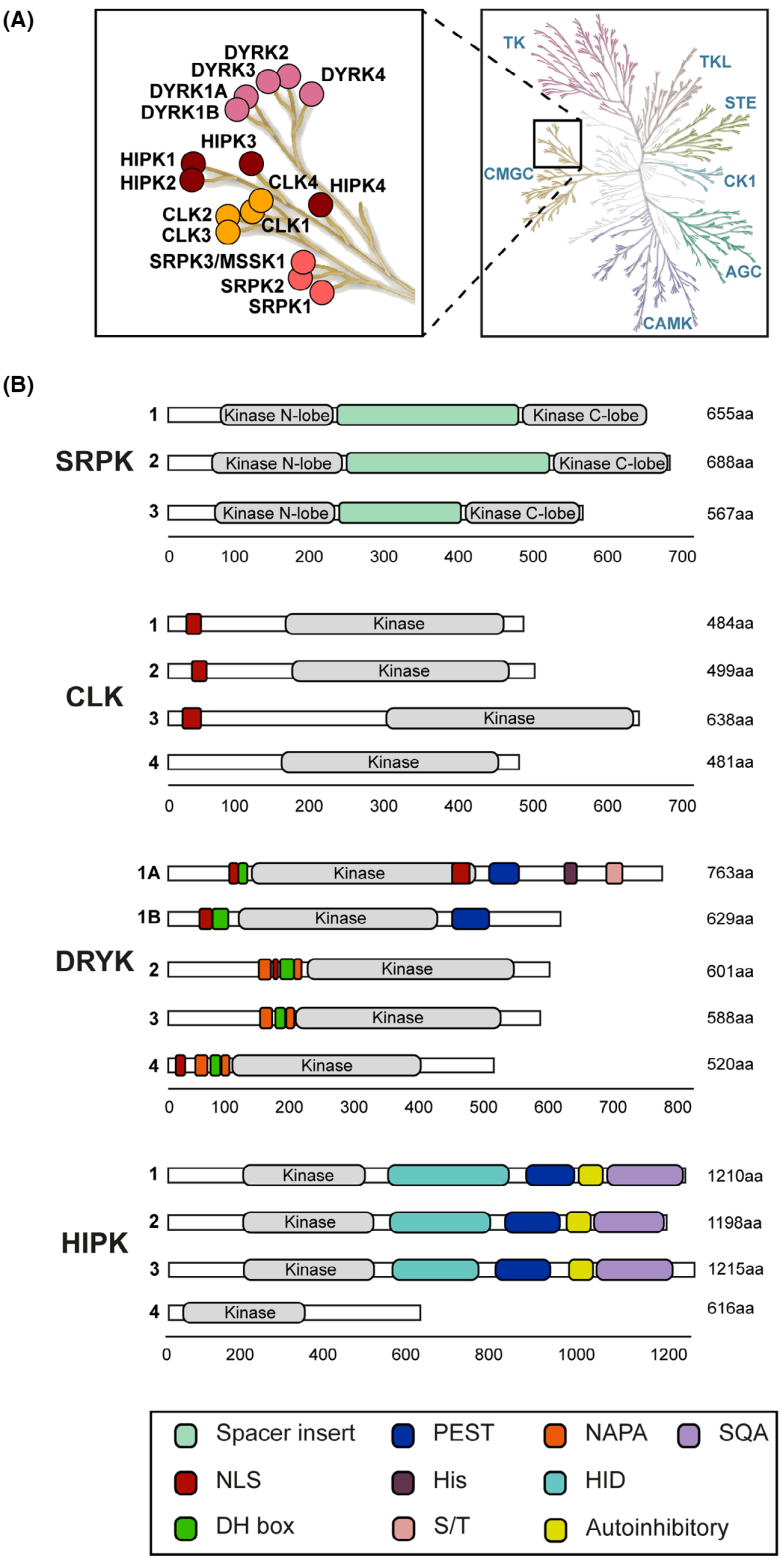
Phylogeny and features of the related SRPK, CLK, and DYRK kinase families. (A) A major branch of the CMGC kinase group contains the kinase families SRPK, CLK, DYRK, and the DYRK sub‐family HIPK. Figure reproduced from kinmap software [323] reproduced courtesy of Cell Signalling Technology, Inc. (www.cellsignal.com) (B) Schematic of the molecular features of human kinases with domains and key regulatory motifs indicated.

Protein kinases are versatile machinery acting within tightly balanced signalling networks. In the field of embryonic stem cells and development, whilst a wealth of knowledge exists and research continues to explore key transcriptional networks, the role and functional importance of protein kinases in development are becoming increasingly understood [1]. The CMGC protein kinase group (named after CDKs, MAP kinases, GSKs, and CDK‐like kinases) comprises several of the best‐studied protein kinases and includes nine kinase families that are predominately proline‐directed serine/threonine kinases and have been extensively reviewed elsewhere [2, 3, 4, 5, 6, 7, 8]. However, in recent years, there has been increased research into a relatively lesser‐studied branch of the CMGC group comprising three kinase families, on which this review will focus: Serine/arginine protein kinases (SRPKs), CDC2‐like kinases (CLKs), and dual‐specificity tyrosine‐regulated kinases (DYRKs), which comprise DYRK1, DYRK2, and homeodomain‐interacting protein kinase (HIPK) sub‐families. Whilst these kinases were initially identified and characterised largely *via* detailed yeast and fly studies, their conservation and expansion in higher organisms means they are represented by increasingly diverse family groups, providing both nuance and redundancy in mammals, which has hampered efforts to elucidate function. However, evidence is mounting for key functional roles in human development and disease. This includes established connections such as DYRK1A in Trisomy 21/Down's syndrome and intellectual disability, and more novel identifications of SRPK signalling and mutations in intellectual disabilities. We conclude that this branch of the CMGC kinase family (Fig. 1) plays important roles in the regulation of developmental processes, such that the signalling pathways in which these kinases operate are found to be disrupted in patients with developmental disorders.

## Identification, structure and catalytic mechanisms of SRPK, CLK, DYRK and HIPK kinases

### The SRPK family

**Fig. 2:**
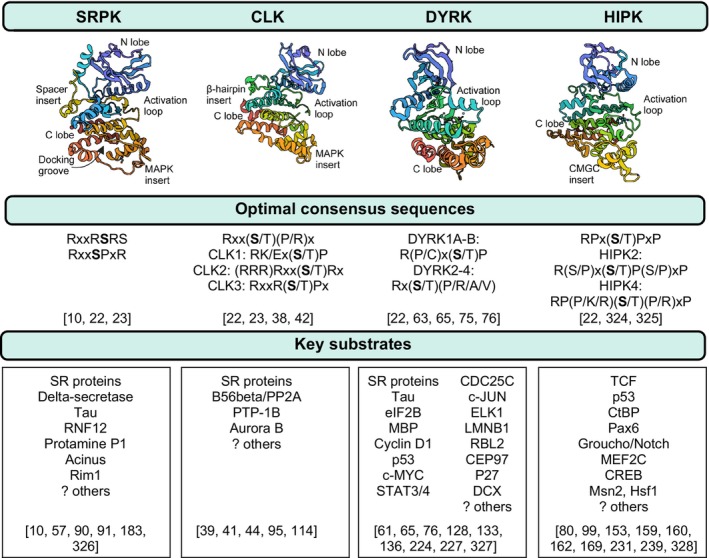
Biochemical characteristics of the SRPK, CLK, and DYRK kinase families. Key structural features, motifs, and known substrates for the related SRPK, CLK, and DYRK kinase families and HIPK sub‐family. Lists of substrates typically refer to studies in mammalian systems except for HIPK, which includes direct substrates identified from *Drosophila* studies. PDB IDs: SRPK1 (1WAK), CLK1 (6KHD), DYRK1A (7AJ2), HIPK2 (7NCF) [10, 22, 23, 38, 39, 41, 42, 44, 57, 61, 63, 65, 75, 76, 80, 90, 91, 95, 99, 114, 128, 133, 136, 153, 159, 160, 162, 169, 183, 224, 227, 231, 239, 324, 325, 326, 327, 328]. Created with Biorender.com.

The serine/arginine (SR) rich splicing factor (SRSF) protein kinase (SRPK) family (Fig. 2) comprises three members, SRPK1‐3. Research has focused on SRPK1 as the prototypical member first identified in the mid‐1990s as a kinase that phosphorylates serine/arginine (SR)‐rich proteins in the regulation of mRNA splicing [9, 10]. SRPK2 was subsequently identified in mouse and human foetal brain [11, 12], with SRPK3 initially named as the protein‐coding gene *Stk23* and identified as a muscle‐specific serine kinase (MSSK1) [13, 14].

SRPKs are characterised by a bipartite kinase domain, with the N‐ and C‐ lobes connected by a disordered spacer insert thought to stabilise the conformation of the kinase lobes [15, 16]. This spacer also influences SRPK localisation, whereby deletion induces a translocation to the nucleus [17, 18], and also affects stability in the case of SRPK1 [19]. However, the spacer insert is not critical for catalytic activity, so long as the N‐terminus remains intact to stabilise the activation loop [20]. In contrast to many other mammalian kinase families, SRPKs are considered to be constitutively active; both SRPK1 structures in the presence and absence of substrate peptide are highly superimposable, and mutations disrupting the contacts between the activation loop and kinase lobes of SRPK1 do not significantly affect activity, indicating that the activation loop is stably in the active conformation [20]. SRPKs auto‐phosphorylate, although this is not a requirement for full activity but may influence sub‐cellular localisation. Upon Epidermal Growth Factor (EGF) stimulation, activated AKT induces SRPK1/2 auto‐phosphorylation at T326 and S587. This drives enhanced Hsp70/90 chaperone‐mediated localisation of SRPK1/2 to the nucleus, followed by hyper‐phosphorylation of SR‐rich proteins and splicing activity [21].

With a preference for serine over threonine [11], SRPKs characteristically phosphorylate regions of repeated arginine/serine (RS) motifs, or serines with arginine at positions P − 3 and P − 2, in addition to arginine or proline at P + 1 [10, 22, 23]. SRPKs contain a mitogen‐activated protein kinase (MAPK) insert that, with helix αG, creates a deep acidic substrate docking groove outside the ATP binding region in the C‐lobe. This groove is a characteristic feature of these kinases, enabling proper binding and sequential phosphorylation of basic repetitive RS regions in substrates [16, 24]. For SRPK1, there is evidence that the kinase phosphorylates in a processive manner; start‐trap experiments, in which a peptide inhibitor is used to block dissociated kinase but fails to block an enzyme processing along a given substrate, elegantly show the processive nature of SRPK1 phosphorylation [25].

The canonical substrates of SRPKs are SR‐rich proteins, most notably the SR splicing factor proteins (SRSFs). SRSFs contain RNA recognition motifs and RNA binding domains and are characteristically enriched in repeated RS sequences in the RNA recognition motifs (known as the ‘RS domain’). The phosphorylation of SRSF proteins by SRPKs has been definitively demonstrated, where phosphorylation of the SRSF1 (ASF/SF2) and SRSF2 (SC35) RS domains by SRPKs induces nuclear import and binding to the U1 small nuclear ribonucleoprotein (snRNP)‐specific 70‐kD protein (U1‐70K) to promote splicing [11, 26]. This is a conserved mechanism and function, as mammalian SRPK1 or CLK1 can restore the interaction of SRSF1 and SRSF2 with U1‐70K in yeast lacking the SRPK orthologue Sky1p [26].

### The CLK family

Like SRPKs, the highly conserved CLK1‐4 family was identified as splicing‐specific kinases, regulating mRNA processing and progression through the cell cycle [27, 28, 29, 30, 31, 32]. The CLK orthologue in *Drosophila* (*Doa*) and its paralogues were classified as members of the LAMMER protein family (by the presence of a conserved EHLAMMERILG sequence) and form the basis of many structural and functional studies [33, 34, 35]. Mammalian CLK1 was identified using a phosphotyrosine antibody screening approach on a cDNA expression library, followed by validation of mRNA transcript levels from mouse embryonic carcinoma cells [31]. These screening experiments and others identified that CLKs are dual‐specific with a propensity to auto‐phosphorylate serine, threonine, and tyrosine residues *in vitro* [27, 30, 31, 32]. As with SRPKs, there are indications that auto‐phosphorylation contributes to CLK localisation [36, 37], but auto‐phosphorylation in the N‐terminal regions is unlikely to be required for either activity or substrate binding *in vivo* [36, 38]. However, CLK auto‐phosphorylation may affect the dimerisation of alternative spliced CLK variants, although the functional relevance remains to be elucidated [37]. Furthermore, the role of low‐stoichiometry tyrosine auto‐phosphorylation *in vivo* remains uncertain [35, 36].

CLKs contain an arginine/serine (RS) domain close to the N‐terminus, followed by a disordered region, a contiguous kinase domain (not bipartite, as found in SRPKs), with a MAPK insert, but lack an SRPK‐like acidic docking groove [24]. Like other splicing kinases, CLKs phosphorylate SR proteins, but with broader substrate specificity compared to SRPKs, *via* additional recognition and phosphorylation of SP and SK dipeptides [23, 38, 39, 40, 41]. The relaxed constraints on substrate motif compared to SRPK are thought to result from helix αH in the kinase C‐lobe blocking formation of the SRPK‐like substrate docking groove, thereby altering charge dispersal in the CLK C‐lobe and removing a preference for basic substrates as seen in SRPKs [42]. Additionally, CLKs have a short β‐hairpin insert at the top of C‐lobe where the large disordered spacer insert is in SRPKs, which forms a hydrophobic patch near the hinge region of the kinase, although how the β‐hairpin insert affects substrate specificity is not fully determined [42]. CLK phosphorylation of splicing factors impacts function, as exemplified by SRSF1, in which CLK1 can phosphorylate three critical SP dipeptides in the RS domain of SRSF1, leading to conformational changes in SRSF1 and dissociation from nuclear speckles [43].

An interesting feature of CLK signalling and function is autoregulation of its own pre‐mRNA maturation and alternative splicing in stress‐responsive, cell‐cycle‐dependent, and temperature‐dependent manners [44, 45, 46]. Most notably, CLK1 and CLK4 were found to be ubiquitylated and degraded following auto‐phosphorylation, whereby inhibition of CLK1 (and CLK4) increased CLK1 protein levels. Moreover, the decrease in CLK activity following inhibition leads to reduced *CLK1* exon 4 skipping and reduced production of truncated (exon skipped) variants [44, 47, 48]. Similarly, exon skipping has been shown to occur under temperature‐sensitive conditions, particularly controlled by CLK1 and CLK4, which appear to be conserved mechanisms across several reptile and mammalian species [45]. High temperatures lead to inactive CLK1/4, reduced SR protein phosphorylation, reduced exon skipping, and alternative splicing, subsequently altering gene expression profiles [45, 46].

### Interplay between SRPK & CLK kinases

Mechanistic dissection of SRPK/CLK kinase‐substrate relationships found that phosphorylation of an SR protein could ‘prime’ the RS domain for subsequent phosphorylation by SRPK and CLK kinases, and that SRPKs and CLKs are regiospecific in substrate phosphorylation. SRPK1 preferentially contacts the N‐terminal region of the SRSF1 RS domain, phosphorylating up to the first 12 serine residues, whereas CLK1 can phosphorylate all serines within an RS domain [40, 49]. Upon SRPK1 binding to the basic region on a given substrate, phosphorylation occurs in a processive ‘directional sliding’ manner away from the acidic SRPK substrate docking groove, as exemplified by the SRSF1 RS domain [50]. In contrast, as discussed above, CLK differs in docking and charge dispersal, where the lack of the acidic SRPK‐like substrate docking groove enables CLK to phosphorylate indiscriminately on substrates primed by SRPK [42]. In addition to a temporal priming relationship, direct interplay between SRPK and CLK has been suggested, where a bridging motif between the CLK1 N‐terminus and a charged surface on SRPK1 forms to stabilise an SRPK1‐CLK1 complex [51].

The cellular localisation of SR proteins dictates their function as regulators of alternative splicing and mRNA export, whereby cytoplasmic phosphorylation of the RS domains is required for nuclear import *via* increasing affinity to transportins [26, 52, 53, 54]. The subsequent nuclear distribution of SR proteins to nuclear sub‐compartments known as ‘nuclear speckles’ [55] is further controlled by co‐ordinated phosphorylation by CLK and SRPK. SRPK dissociation from chaperones facilitates its own transport to the nucleus and notably to these nuclear speckles [17, 21, 24, 40, 52, 53]. After nuclear import, SR proteins concentrated in nuclear speckles undergo further hyper‐phosphorylation by CLK and SRPK. This leads to conformational change, driving diffusion into the nucleoplasm and the catalysis of pre‐mRNA splicing at 5′ splice sites by modulating the assembly of spliceosome machinery, including U1‐snRNP and U4/U6‐U5 tri‐snRNPs [24, 40, 43, 56, 57]. Furthermore, SRPK and CLK can form a complex to promote the hyper‐phosphorylation and release of SR proteins out of nuclear speckles to affect gene‐specific alternative splicing patterns [51].

### The DYRK kinases

The DYRK kinases (DYRK1A‐B, DYRK2‐4) were initially named as the Yak subgroup in budding yeast (Yak1p) [58], with the *Drosophila* orthologues named *minibrain* (*mnb*, DYRK1A/B) due to an ascribed developmental defect of *mnb* mutant files [59] and *Dyrk2‐3* (DYRK2‐3). DYRKs were established in the late 1990s as a distinct kinase subfamily [60, 61], and following identification in multiple organisms, DYRK1A is the most researched kinase of the families discussed in this review.

The structural features of DYRKs diverge this family away from the closely related SRPK and CLK families (Fig. 1). In addition to the kinase domain, DYRKs contain one or two nuclear localisation signals (NLS), a DYRK homology box (DH domain), a PEST sequence (for proteolytic degradation), and an N‐terminal auto‐phosphorylation accessory region (NAPA). DYRKs are further classified as Class I (DYRK1A‐B) and Class II (DYRK2‐4) reflecting their regulation and structural features. For example, the NAPA domain is not present in Class I DYRK1A‐B but is essential for catalytic activation in Class II DYRK2‐4 [62]. The DH box plays a minor role in DYRK maturation and auto‐phosphorylation alongside the NAPA domain, assisting folding and stability of the nascent protein kinase domain [63, 64]. Finally, the NLS is not present in DYRK3 and DYRK4 due to alternative splicing [65].

Work on the DYRK catalytic mechanism has roots in *Drosophila* studies of *mnb* [62, 66] and orthologues in *Saccharomyces pombe* (Pom1p) and *Saccharomyces cerevisiae* (Yak1p) [67, 68]. DYRKs require activation loop phosphorylation for full activity [60, 61, 69], and recent work has revealed a requirement for proline hydroxylation of DYRK1A and 1B at an L/xGxP motif by 2‐oxoglutarate‐ and oxygen‐dependent dioxygenase PHD1, prior to tyrosine auto‐phosphorylation for full catalytic activity. Interestingly, this has been proposed as a general mechanism of CMGC kinase activation [70].

Mammalian DYRKs have dual specificity, whereby they can phosphorylate serine, threonine, and tyrosine, although tyrosine phosphorylation appears to be restricted to auto‐phosphorylation for catalytic activation [66]. This contrasts with CLKs, where the function of tyrosine auto‐phosphorylation remains unclear. Interestingly, DYRKs typically act as priming kinases, whereby they phosphorylate substrates to facilitate the recruitment of other kinases for further phosphorylation. This parallels the priming of SR proteins by SRPK for subsequent CLK binding and phosphorylation. The prototypical example for DYRKs is priming another CMGC kinase, GSK3β, to phosphorylate diverse substrates such as NFAT1, eIF2Bɛ, Tau, and MAP1B [71, 72, 73, 74].

Subtle differences in consensus motif between DYRK family members have been found. Whilst there is ostensibly a requirement for proline at the plus one position (P + 1) to the phospho‐serine and arginine at P − 3, both valine and alanine at P + 1 can be tolerated [63, 75, 76]. DYRK1A requires arginine at P − 3 and proline at P + 1, although proline at P − 2 may also be important (RPXS/TP) [75]. DYRK2 and 3 can instead tolerate arginine at P − 2 (instead of P − 3), and do not require proline at P − 2 (RXS/TP) [76]. Furthermore, *in vitro*, DYRK2 can phosphorylate peptides with valine at P + 1 to a greater extent than DYRK1A or DYRK3 [76]. This may explain why some substrates (such as Histone H2B) can be differentially phosphorylated by the DYRK family [61] and the absence of proline at P + 1 should not be used to exclude potential candidates [77]. Moreover, a peptide microarray analysis similarly identified DYRK2 and DYRK4 as less dependent on arginine at P−2/−3 than DYRK1A, which may enable phosphorylation of a more diverse sub‐set of peptides compared to DYRK1A, including peptides on candidate substrates such as c‐JUN, STAT4, ELK1, CDC25C, MYC, p53, and PRKAA1 [22, 65].

### The HIPK kinases

HIPK is a closely related DYRK sub‐family with overlapping structural features [78]. HIPK1‐3 were identified in the 1990s [79], with HIPK4 characterised more recently. HIPK2 is the prototypical family member and the best‐studied, whilst HIPK4 is the least conserved and found only in mammals [80], suggesting a divergence in function from HIPK1‐3. HIPKs contain a kinase domain, a homeobox‐interacting domain for protein–protein interactions (HID; HIPK1‐3 only), PEST domain (HIPK1‐2 only), an autoinhibitory region (identified in HIPK2 [81]), and disordered serine (S), glutamine (Q), and alanine (A) repeats at the C‐termini (also known as the tyrosine (Y) histidine (H) rich region). Similarly to DYRKs, auto‐phosphorylation is required for maximal catalytic activity [82]. Notably, HIPK2 auto‐phosphorylation at the activation loop (Y354/S357) and T880/S882 in response to DNA damage allows for PIN1 binding [83, 84], which stabilises HIPK2 by conformation change and dissociating HIPK2 from the E3 ubiquitin ligase SIAH‐1. Stabilisation of active HIPK2 by PIN1 also increases its affinity for substrates and promotes its apoptotic functions in DNA damage‐stressed cells [83, 84]. Auto‐phosphorylation also leads to re‐localisation from speckle‐like regions to the cytoplasm for HIPK1 and 2, but not 3 and 4 [85]. The C‐terminal region (overlapping with the PEST sequence) may also be important for localisation to nuclear speckles, whereby HIPK2 is SUMOylated, and this modification by SUMO‐1 together with the E2 conjugating enzyme UBC9 directs HIPK2 localisation to nuclear speckles [86].

### Summary

Whilst there are similarities in the structural features and mechanistic functions discussed above between SRPK/CLK/DYRK kinase families, there is divergence in the phylogeny of SRPK/CLK from DYRK/HIPK that is reflected in the range of structural features and domains (Fig. 1), substrate recognition motifs, and key kinase substrates (Fig. 2). Further structural differences between SRPK and CLK enable their unique yet overlapping functions in the co‐ordinated phosphorylation of SR proteins. The broad similarities in consensus motifs (arginine and serine rich) further dictate this activity, with subtle differences indicative of additional specific functional roles for SRPK and CLK. In contrast to SRPK/CLK, DYRK and HIPK are proline‐directed kinases that require auto‐phosphorylation for maximal kinase activity and do not predominately phosphorylate within repetitive arginine and serine domains. These differences in structure and consensus motifs within and between the SRPK/CLK/DYRK family members reflect the wide variety of identified substrates to date. Differential functions of these kinases will be further conferred by subcellular localisation and cell type specific expression.

## Functions of SRPK, CLK, and DYRK kinase family members

CMGC protein kinases of the SRPK, CLK, DYRK families and the HIPK sub‐family are well studied with respect to their role in phosphorylating and controlling functions of substrates that regulate various aspects of RNA processing and gene expression [2, 3, 87]. These include expression, activity, and localisation of RNA‐binding proteins, spliceosome assembly and alternative splicing, and RNA export and polyadenylation. Spatial restriction of the kinases between the cytoplasm, nucleus, and sub‐nuclear compartments (i.e. speckles) acts as a mechanism for controlling substrate specificity and cellular function in response to specific cues. For an overview of the key cellular functions associated with the related SRPK, CLK, and DYRK kinase families, refer to Table 1.

**Table 1:** Splicing and signalling functions associated with SRPK, CLK, and DYRK kinase families. Splicing functions and signalling pathways associated with the activity and/or presence of the related SRPK, CLK, and DYRK kinase families and the HIPK sub‐family in various organisms. MEFs, mouse embryonic fibroblasts; SR, serine/arginine‐rich proteins. Representative references. Created with BioRender.com.

	Splicing regulation	Signalling	Ciliogenesis, cell cycle and stress response
	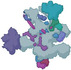	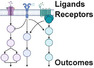	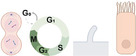
SRPK	Splice site regulation [106] SR protein phosphorylation, nuclear import, and dispersion of nuclear speckles [17, 26, 40, 49, 50, 53, 329, 330] Splicing of non‐consensus introns [331] Spliceosome stabilisation [57] Lipogenic gene splicing and cell growth [288, 307]	p‐AKT regulation in MEFs and transformation in mice [113] mTORC1‐S6K1 and hexosamine biosynthetic pathways [288, 307] SR protein phosphorylation and CGG repeat RNA‐induced neurodegeneration [332] Delta secretase and tau phosphorylation [91, 92]	IGF1/S6K1 and stress granule formation [111]
CLK	SR protein phosphorylation, nuclear import, and dispersion of nuclear speckles [17, 40, 43, 49, 50, 53, 329, 330] Splicing control and intron retention in mESCs [188] Splicing control in stress, cell‐cycle, and temperature‐sensitive manners [44, 45, 46, 47, 48]	ERK1/2‐RSK signalling cascade [189] Fatty acid oxidation gene control in hepatocytes [333] Insulin response through AKT‐PP2A complex regulation [114]	Abscission checkpoint regulation by phosphorylation of Aurora B and B56beta [44, 95]
DYRK	SR protein phosphorylation to regulate splicing [115, 116, 117, 118, 119]	Prime substrates of GSK3β [71, 72, 73, 74] Erythropoiesis and haematopoietic progenitors [206] NFAT transcriptional response [207] EGFR signalling [214] Neuronal RAS/B‐RAF/MEK [142] p53 phosphorylation in embryonic cortical neuron precursors [131] Phosphorylate p27, Cyclin D1, and HDAC5/9, contributing to myoblast differentiation [128, 201] Stabilise 4e‐bp1 and induce autophagic flux in Xenopus [202] Complex with E3 ligases [88, 141, 143, 144]	Protein turnover during cell cycle [128, 134, 135, 136, 137] Nuclear export and aggregation of cyclin D1 in Neuro2A cells [217] Ciliogenesis, assembly, morphology, Hedgehog signalling and tissue development [143, 144, 203] Centriole disengagement [227] Organellar phase‐transition and stress granule formation [139, 140] DNA damage response [138]
HIPK		Actin regulation in MEFs [233] Regulate phospho‐SMADs in TGFβ and BMP pathways [252, 254]. ER‐stress response *via* IRE1a‐ASK1‐JNK [279]	UV stress‐induced apoptosis [249] DNA damage response and apoptosis [83, 84, 153, 154] Non‐apoptotic functions in cytokinesis regulation [96, 97, 98]

SRPKs and CLKs are widely considered splicing‐specific kinases; DYRKs have key involvement in the regulation of cell fate and differentiation and the control of substrate turnover; whilst HIPKs act as co‐repressors for homeodomain‐containing transcriptional regulators and are considered regulators of growth and apoptosis. Indeed, comprehensive kinase‐protein interaction landscape studies using affinity‐purification mass spectrometry have identified many SRPK, CLK, DYRK, and HIPK substrates and protein‐interacting partners, notably in the regulation of RNA splicing, transcription, translation, ribosome biogenesis, ribosomal RNA processing, and localisation [88, 89]. Importantly, many other interacting proteins were identified in this study, which may be indicative of ‘non‐canonical’ functions, including DNA damage responses [88]. Further non‐splicing functions of the SRPK family in development and neuropathology have been reported [90, 91, 92, 93]. For CLKs, non‐splicing functions include kinase activation of phosphatases and regulation of cytokinesis [94, 95]. Similarly, non‐apoptotic functions of HIPKs have been described in regulating cytokinesis [96, 97, 98], alongside facilitation of p53‐independent apoptosis [99]. Together, these suggest exciting opportunities to identify novel functions and biology that are controlled by these interesting kinase families.

### Splicing‐related functions of SRPK and CLK

As discussed earlier, serine/arginine (SR)‐rich protein phosphorylation regulates intranuclear movement, distribution, and pre‐mRNA splicing functionalities. SRSF1 is the prototypical SRPK/CLK substrate, whose phosphorylation regulates protein localisation, splicing recognition, and spliceosome assembly [56, 100, 101, 102]. SR proteins have been found to be concentrated in ‘nuclear speckles’; sites for splicing factor storage, modification, and reassembly of splicing factors. Phosphorylated SR proteins diffuse into the nucleoplasm, and associate with pre‐mRNA through their RNA recognition motifs, and bind to small nuclear ribonucleoproteins (snRNPs) to establish the 5′–3′ splice sites and larger spliceosome complexes [101, 103]. SR proteins have also been shown to facilitate the export of mRNA from the nucleus [104]. Cycles of SR protein phosphorylation *via* SRPKs and CLKs regulate spliceosome assembly and activity in the nucleus by disassembly of nuclear speckles [9, 10, 11, 12, 105].

Identification of SRPK and CLK as splicing‐factor kinases responsible for phosphorylation and activation of SR proteins and resulting pre‐mRNA regulation arose from phosphopeptide mapping, mitotic extract phosphorylation reactions, and *in vitro* kinase assays [9, 10, 32, 39]. For example, 3′ splice site regulation was found to be partially dependent on the SRPK1 homologue Sky1p in yeast [106]. Furthermore, SRPK2 is essential for HeLa cell survival through stabilising the integration of the snRNP complex U4/U6‐U5 tri‐snRNP with the spliceosome, which maintains splicing function [57]. Indeed, from a high‐throughput affinity‐purification mass spectrometry study, over half of the identified interactors of SRPKs and CLKs were classified in the ‘regulation of nucleic acid metabolism’ ontology group, with SRPKs having many more interacting proteins involved with RNA processing than other CMGC kinases [89].

Non‐catalytic functions of nuclear SRPK1 in splicing regulation have also been proposed. The SRPK1 spacer insert binds the N‐terminus of the snRNP U1‐70K, facilitating allosteric changes in U1‐70K that alter its binding to associated SR proteins (such as SRSF1) and the selection of specific exonic splicing enhancers (ESEs) on pre‐mRNA. This occurs in an ESE‐dependent manner, either promoting or repressing alternative splicing of different downstream mRNAs [107]. CLK1 also binds U1‐70K, but at the C‐terminal region, phosphorylating U1‐70K at S226, which is required for SR protein binding to the snRNP. However, CLK1 binding occurs independently of the SRPK1 spacer insert regulation of pre‐mRNA ESE recognition by snRNP:SR protein complexes, suggesting different functions of these kinases in U1‐70K snRNP complex formation and ESE recognition [107].

SRPK and CLK have been associated with further splicing‐related functions. As discussed previously, these kinases are themselves alternatively spliced [37], and a comparison of full‐length and truncated CLK1 highlighted a CLK1 auto‐regulation splicing loop [108]. A further example includes SRPK phosphorylation activating Cleavage and Polyadenylation Specificity Factors CPSF6/7, which interferes with CPSF6/7 interaction with a subunit of the alternative polyadenylation regulator CPSF, leading to subsequent nuclear localisation and activation [109, 110].

### Novel non‐splicing functions of SRPK and CLK

Although much research on SRPK and CLK has focussed on splicing regulation, there are indications that SRPK and CLK kinases regulate signalling cascades with functions unrelated to splicing [90, 91, 92, 94, 111]. A molecular basis is provided by the identification of > 100 SR proteins in humans and from a computational analysis of mouse cDNAs, which identified 112 conserved arginine/serine (RS) domain‐containing proteins [112]. Of these, approximately 1/3 have known roles in splicing or tissue‐specific alternative splicing, which presents potential for non‐splicing functions of the remaining SR proteins. A key example of a splicing‐independent SRPK kinase function was discovered in SRPK1 homozygous knockout mouse embryonic fibroblasts [113]. SRPK1 can act as a tumour suppressor by forming a complex with active AKT1 and the PHLPP1 phosphatase (alongside chaperones), which together inactivate AKT by dephosphorylating key regulatory sites. Therefore, SRPK1 depletion reduces the recruitment of PHLPP1 to AKT1, leading to increased AKT phosphorylation and activity and cellular transformation in mice. Curiously, overexpression of SRPK1 was also found to be tumorigenic, as high levels of SRPK1 sequestered PHLPP1 away from phosphorylated AKT, driving increased cellular AKT activity [113].

CLKs have also been implicated in splicing‐independent mechanisms of cell cycle regulation. Inhibition of mammalian CLK1, 2, and 4 (using the small molecule inhibitor TG003 or siRNA‐mediated depletion) modulates alternative splicing events and reversal of some CLK‐mediated oncogenic alternative splicing, but also cell division defects [44]. Following CLK inhibition, a reduced proportion of the treated cell population correctly completed cytokinesis, with premature passing of the abscission checkpoint, rapidly broken chromatin bridges, a reduced frequency of midbody formation, and DNA damage [44, 95]. Mechanistically, cell cycle regulation by CLKs is at least in part splicing‐independent, as CLK1, 2, and 4 were shown to directly phosphorylate Aurora B and B56β (a regulatory subunit of the PP2A phosphatase complex) to delay the abscission checkpoint [95]. CLK2 phosphorylation of B56β is further connected with attenuation of AKT signalling, where insulin‐induced AKT activation leads to increased CLK2 expression and stability, and in a negative regulation loop, CLK2 activates the PP2A complex to de‐phosphorylate AKT, coinciding with the highest levels of CLK2 during the cell cycle [114]. Furthermore, the majority of CLK‐dependent alternative splicing events are cell cycle‐regulated, suggesting that CLK activity is coupled to cell cycle progression [44], which may connect CLKs to phenotypes of dysregulated cell division, such as developmental disorders and oncogenic transformation.

### DYRK functions in proliferation, survival, cell cycle, ubiquitin‐mediated degradation of targets, and liquid‐phase transitions

DYRK family kinase functions reported to date mostly involve regulation of cell cycle, proliferation, differentiation, DNA damage, and protein turnover, which is reviewed in [4, 77]. DYRKs were historically classified as specifically cytosolic or nuclear from overexpression studies [61], but this is now more nuanced, with both cytosolic and nuclear localisation and functions ascribed to DYRK isoforms. Similarly to SRPKs and CLKs, DYRK1A can accumulate in nuclear speckles *via* a His‐rich localisation sequence, whereas DYRK1B remains more diffuse across the nucleus [115]. DYRK1A has also been shown to phosphorylate serine/arginine (SR)‐rich proteins including SF3B1, SRSF1, and SRSF2 to regulate splicing, for example, of the mRNA encoding the microtubule‐binding protein Tau [115, 116, 117, 118, 119]. DYRK3 is predominately nuclear, with expression in testes and erythroid cells [120, 121].

A range of functions for the DYRK family have been suggested from genetic studies in nematodes, including regulation of cell cycle, cytokinesis, cell differentiation, and nutrient signalling and sensing. The DYRK2‐3 ortholog in *Caenorhabditis elegans*, MBK‐2 (alongside other kinases such as CDK‐1 and GSK‐3), is required for oocyte‐embryo transition as it marks the RNA‐binding zinc‐finger protein OMA‐1 for ubiquitin‐mediated degradation. Mutations in *mbk‐2* prevent OMA‐1 degradation after meiosis, leading to failure of mitosis and embryogenesis [72, 122, 123]. Interestingly, *mbk‐2* knockout organisms are inviable due to a failure to complete cytokinesis and therefore embryonic development, whereas loss‐of‐function mutants of its paralogue *mbk‐1* (DYRK1A‐B orthologue) are viable [124]. The phenotypic contrast between MBK‐1 and MBK‐2 indicates isoform‐specific functions, which may be reflected in human DYRKs.

A key role for DYRKs is the regulation of cell cycle dynamics, predominately through the control of protein turnover and induction of ubiquitin‐mediated degradation of targets, as reviewed in [4]. Therefore, DYRKs are thought of as potential targets in cancer, as reviewed in [125, 126]. DYRK1A (and also DYRK1B and DYRK2) can negatively regulate protein levels of Cyclin D1‐3 and MYC but positively regulate others, including p21, p27, p53, and the DREAM complex subunit Lin52 [127, 128, 129, 130, 131, 132]. DYRK1B can induce cell arrest in the G1 phase by priming Cyclin D1 for further phosphorylation by GSK3β and targeting for proteasomal degradation, whilst p27(kip1) is stabilised [133, 134]. Similarly, DYRK1B primes the ubiquitylation and degradation of c‐JUN and c‐MYC transcription factors for negative regulation of the G1/S transition [128, 135, 136]. Furthermore, during the cell cycle or in response to DNA damage, DYRK2 can phosphorylate CDC25A to induce its ubiquitin‐mediated degradation, delaying mitotic exit, whilst, in a mutual negative feedback mechanism, CDC25A can dephosphorylate DYRK2 to control its localisation and activity towards other substrates [137]. Indeed, cytosolic DYRK2 can accumulate in the nucleus in response to DNA damage or genotoxic stress, by increasing the interaction and phosphorylation of p53 at S46 [138]. These functions of DYRKs in promoting and delaying cell cycle progression reflect isoform differences in substrates and highlight how alterations in activity or expression may influence cell cycle control.

DYRK3 may also contribute to the regulation of mitosis and stress responses by altering subcellular liquid‐phase transitions. DYRK3 colocalises with nuclear speckles and centrosomes, especially when inactive or inhibited, and the active kinase has been implicated as a ‘dissolvase’ of these membraneless organelles during mitosis [139]. This is thought to be due to an increase in DYRK3 interaction with and phosphorylation of RNA‐binding proteins, which are core components of nuclear speckles [139]. Similarly, DYRK3 can control the dissolution of stress granules, whereby the N‐terminal region of inactive DYRK3 prevents the dissolution of stress granules, blocking the activity of signalling components such as serine/threonine‐protein kinase mTORC1. However, when DYRK3 is active, it both promotes the dissolution of stress granules and the phosphorylation of the mTORC1 negative regulator PRAS40, which drives mTORC1 re‐activation following stress [140].

Interestingly, although DYRK functions are predominantly kinase activity‐dependent, scaffolding functions have also been proposed, particularly within E3 ubiquitin ligase complexes [141, 142]. DYRK2 acts as an adaptor protein within the EDVP E3 ligase complex (containing EDD, DDB1, and VPR Binding Protein), where the DYRK2 catalytic activity is required for substrate ubiquitylation‐degradation but not for complex formation itself [88, 141, 143, 144]. Furthermore, DYRK1A and DYRK1B can interact with adaptor proteins DDB1‐ and CUL4‐associated factor 7 (DCAF7/WDR68/HAN11) of the CUL4‐DDB1 E3 ubiquitin ligase [145, 146]. DYRK1A interaction with DCAF7 appears to modulate function by inducing DCAF7 nuclear localisation [147], which may negatively regulate GLI1 transcriptional activity [148], important in Hedgehog developmental signalling. Finally, in zebrafish, the interaction of dyrk1b and the E3 ubiquitin ligase wdr68 is thought to be essential for control of endodermal development and correct craniofacial patterning [149].

### HIPKs function in stress response and transcriptional regulation

HIPKs are activated in response to several cellular stressors and are generally considered regulators of growth and apoptosis, with established roles (particularly for HIPK2) as regulators of developmental signalling and differentiation (e.g., Wnt, Shh, JAK/STAT, Hippo, and JNK). HIPKs are predominately nuclear kinases, translocating to the nucleus following cellular stress to function as co‐repressors of homeodomain‐containing transcriptional regulators, although cytoplasmic activity has also been reported [79, 150, 151]. HIPKs phosphorylate several proteins, including p53, NKX1.2, and the androgen receptor, and similar to DYRKs, are involved in cellular protein turnover and stability [4]. HIPK2 is the best‐characterised member of the HIPK sub‐family, with a role in p53 activation in response to DNA damage [152, 153, 154]. Indeed, HIPK2 has been shown to be regulated by the ATM/ATR checkpoint kinases, and its turnover is controlled by E3 ubiquitin ligases including SIAH‐1, SIAH‐2, WSB, MDM2, and SCFFbx3 [155, 156, 157, 158]. Furthermore, in response to arsenic‐induced cell stress, HIPK2 increases phosphorylation of cAMP‐responsive element‐binding protein (CREB) at S271, contributing to transcriptional activation of genes including CCNA1, SGK1, and CALB1 [159].

As stress‐responsive proteins, HIPKs act to maintain cellular homeostasis. In glucose‐depleted conditions, yeast HIPK (Yak1p) translocates to the nucleus and phosphorylates transcriptional stress factors Msn2 and Hsf1, leading to an increase in heat shock transcription programmes to maintain cellular homeostasis [160]. Similarly, *Hipk3* gene disruption in mice highlights the HIPKs in response to metabolic or glucose stress, whereby pancreatic islets of *Hipk3* ^−/−^ mice display lower levels of insulin secretion and beta cell proliferation than wildtype animals, with the defect in insulin secretion being partially rescued by Wnt3a and a GSK3β inhibitor [161]. HIPK2 can also increase proglucagon promoter activity *via* phosphorylation of transcription factor PAX6, which enhances PAX6 interaction with transcriptional coactivator p300 to promote gene transcription [162]. Further exploration is required to understand the physiological relevance of HIPKs to glucose homeostasis since the major phenotypes of HIPKs in model organisms (discussed later) relate to defects in neuronal, ocellar, and wing development and embryonic lethality.

Like DYRKs, HIPKs can also regulate cell cycles and apoptosis. One mechanism appears to be *via* interplay with p53. HIPK2 phosphorylates p53 at S46 following UV‐induced DNA damage, thereby inducing apoptosis [153, 154]. HIPK4 can phosphorylate p53 at S9, whilst overexpression of HIPK4 decreases expression of Survivin, an anti‐apoptotic gene regulated by p53 [80]. In nuclear bodies, the tumour suppressor protein ProMyelocytic Leukaemia has been characterised as a cofactor for interaction between HIPK2 and p53 [153, 154, 163]. Other cofactors for HIPK phosphorylation of p53 have been identified, including Axin, SP100, Daxx, and p54DINP1 [81, 163, 164, 165]. Intriguingly, HIPK2 can sensitise cells to apoptosis in a p53‐independent manner *via* phosphorylation at S422 of the transcriptional corepressor C‐terminal Binding Protein (CtBP), which leads to the de‐repression of genes encoding BAX/NOXA apoptosis regulator proteins [99, 166].

HIPKs are also implicated in the regulation of mitosis. HIPK2 co‐localises at the midbody with Histone H2B following recruitment by Aurora B and phosphorylates Histone H2B at S14, which is required for successful cytokinesis [96, 98]. Similarly, an alternative isoform of HIPK2 (short ‘‐S’ form, following intron 13 retention and translation of a shorter protein lacking part of the auto‐inhibitory domain) is required for cytokinesis, with depletion of the short form resulting in fewer cells completing abscission, leading to increased binucleation [97].

As alluded to earlier, HIPKs can function in transcriptional regulation. For example, HIPK2 can localise to chromatin and function as a transcriptional coactivator in response to nuclear hormone receptors such as oestrogen [167]. HIPK2 can also form a complex with the co‐repressor Groucho in *Drosophila* and the histone deacetylase HDAC1 to repress gene expression alongside NK homeobox transcription factors [79, 168]. However, activated HIPK2 can also phosphorylate Groucho in a S/P‐rich region, thereby releasing the repressive complex and de‐repressing target genes [169]. Additionally, phosphorylation of Histone H3 by HIPK4 *in vitro* may also suggest a wider role for HIPK in regulating chromatin condensation [80]. Furthermore, in *C. elegans*, the HIPK1 homologue HPK‐1 can indirectly transcriptionally regulate proteostasis as a component of both the heat shock factor protein 1 (HSF‐1) pathway inducing chaperone protein transcription following heat shock and the TORC‐1 pathway inducing autophagy following nutrient depletion [170].

### Summary

The functions of the related SRPK, CLK, and DYRK kinase families cover the breadth of cellular activity (Table 1). The regulation of splicing through phosphorylation of SR proteins by SRPK and CLK is also evident for DYRKs, whereas HIPKs have not yet been reported to directly control SR protein activities. However, these kinases can translocate to the nucleus, where they exhibit further functional overlap besides splicing regulation (SRPK/CLK/DYRK), including regulation of transcriptional elements and cell cycle progression (DYRK/HIPK/CLK). Further cellular activities occur across subcellular compartments (Table 1) including from regulation of AKT signalling (SRPK/CLK) to cellular homeostasis following nutrient stress (DYRK/HIPK) and induction of apoptosis following DNA damage (DYRK/HIPK), with the potential for many more exciting functions to be identified.

An interesting commonality in SRPK, CLK, and DYRK functions is regulation of diverse aspects of the cell cycle. Whilst CLK control of splicing activity is coupled to cell cycle progression, further non‐splicing functions overlap with HIPK activity in the regulation of the abscission checkpoint required for correct cytokinesis. In most cases, catalytic activity is required, although there is evidence of additional scaffolding and adaptor roles for DYRKs and HIPKs in multi‐protein complexes. In particular, DYRKs and HIPKs regulate protein turnover and stability through complexing with E3 ligases, and SRPK is implicated in the control of E3 ligase activity (discussed later [93]); however, as yet, there is no established role for CLK in controlling components of ubiquitin‐mediated signalling. Importantly, there is increasing evidence for non‐splicing functions of SRPKs and CLKs that overlap with DYRK and HIPK functions, particularly in relation to their role during development, which will now be discussed.

## Specific functions of SRPK, CLK, and DYRK family kinases in stem cells and development

SRPK and related kinases perform key functions in signalling pathways and cellular processes related to embryonic development and differentiation (Table 2). The use of genetics in model organisms and stem cells has underpinned our understanding of the role these kinases play in embryonic development (Table 3). Recent research has further identified functions that have interesting implications for our understanding of human development. It is also well established that dysregulated embryonic development and/or alterations in the differentiation of specific cell types and tissues underlie multiple complex developmental disorders. Here, we discuss insights into the functions of the related SRPK, CLK, and DYRK families in stem cells and development, with emphasis on potential roles in neurodevelopmental disorders such as intellectual disability, which can be caused by defects in neural differentiation, neurite outgrowth, migration, and synaptic transmission.

**Table 2:** Functions of SRPK, CLK, and DYRK kinase families in development, neurobiology and fertility. Associations of the related SRPK, CLK, and DYRK kinase families and HIPK sub‐family with stem cell, developmental and fertility functions in various organisms. Representative references. Created with BioRender.com.

	Stem cell regulation and differentiation	Neuronal development and function	Spermatogenesis and fertility
	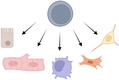	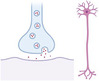	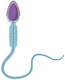
SRPK	Germ cell differentiation [184] Phosphorylation of developmental E3 ubiquitin ligase RNF12/RLIM [93, 178] Developmental angiogenesis in zebrafish [334]	Nervous system integrity, locomotor function, and lifespan [175, 176, 179] Presynaptic active zone organisation, neurotransmitter release, and homeostatic plasticity [181, 182] Expression in a sub‐set of neurons [93, 258] Zebrafish startle response [334, 335]	Phosphorylate protamine to initiate zygotic genome activation and paternal genome reprogramming [90]
CLK	Neuronal differentiation in PC12 cell line [189] Low expression in skeletal and cardiac differentiated PC19 cell line [31]	Xenopus dorsal mesoderm and ectoderm morphology [336] Induction of neural markers modulating BMP signalling in Xenopus [190]	High expression in mature murine spermatozoa [192]
DYRK	Differentiation of osteoclasts [200], myoblasts [128] and skeletal muscle [202] Neural stem cell self‐renewal in response to EGF [214] Skeletal development and ciliogenesis [203] Neural development and differentiation; progenitor proliferative capacity [115, 216, 217, 218, 219, 220] Oocyte‐to‐embryo transition [72, 122, 123]	Neuroblast spacing, brain size and development [59] Asymmetrical division of neural stem cells [214] Neurite outgrowth and branching [224] Murine microcephaly, body weight and motor function [208, 211] Neurobehaviours controlling freeze/startle response [208] Proliferation of embryonic cortical neuron precursors [131]	
HIPK	Repress MEF2‐dependent gene expression in undifferentiated myoblasts [231]	Sonic hedgehog‐induced proliferation of the mesoderm and neural tube [249] Eye, ocellar, and bristle development from the neuroectoderm [256] Maintenance and survival of sensory, sympathetic and dopamine neurons, and glial cell development [248, 250, 251, 252, 253, 254]	HIPK4 levels regulate sperm motility, morphology and oocyte binding capacity [233]

**Table 3:** Key genetic phenotypes of the SRPK, CLK, and DYRK kinase families in model organisms. Summary of key genetic phenotypes (survival/essentiality and neurological) arising from the genetic ablation, knockout, or knockdown of SRPK, CLK, DYRK, or HIPK kinases in model organisms. Information collected from literature and orthologue databases, including *S. cerevisiae* Genome Database, WormBase, MouseMine/MGI, Xenbase, and FlyBase. Created with BioRender.com.

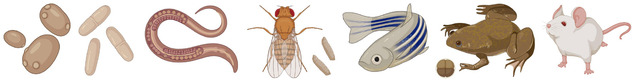
**SRPK**
Not essential in yeast Knockdown in worm is embryonic lethal or with rare surviving sterility Essential in fly, and a neuronal isoform is required for proper motor behaviour Zebrafish *Srpk2* regulates startle response Murine *Srpk1* is essential; *Srpk2* is not essential; *Srpk3* controls cardiac and skeletal muscle growth [14, 18, 113, 173, 174, 175, 176, 177, 334, 335, 337]
**CLK**
Required for splicing in worm Homozygous loss is embryonic lethal in fly; heterozygous loss disrupts neuroectoderm formation and larval segmentation; reduced activity controls male sex determination Regulates embryogenesis, dorsal morphology, and neuroectoderm formation in frog CLK1 affects viral mRNA processing in mice; CLK2 alters fatty acid metabolism; CLK4 protects against cardiac hypertrophy [23, 33, 39, 190, 191, 193, 194, 195, 336, 337]
**DYRK**
Negative regulator of yeast cell growth; confers sensitivity to heat shock Worm DYRK1A‐B orthologue contributes to olfactory sensing Worm DYRK2‐3 orthologue regulates the oocyte‐embryo transition Fly DYRKs affect behaviour, memory, vision, and olfaction Frog *Dyrk1a* controls proper ciliogenesis and brain size Zebrafish *dyrk1aa* contributes to brain size and social behaviour; *dyrk1b* is essential and promotes endoderm formation and craniofacial patterning Murine *Dryk1a* is essential and protects against microcephaly. *Dyrk1b* loss leads to pre‐weaning lethality, and heterozygotes have altered neurological behaviours. *Dyrk2* is essential and controls ciliary morphology and skeletal development. *Dyrk3* and *4* are not essential for viability or fertility [59, 72, 122, 123, 124, 149, 177, 198, 202, 203, 204, 208, 211, 222, 223, 228, 338, 339, 340]
**HIPK**
Accelerates ageing in worms, decreases stress resistance, and is required for normal brood size and germline proliferation Fly *Hipk* is essential in pupae; it is required for wing development, ocellar development, eye size, and the presence of photoreceptors and bristles Zebrafish Hipk2 protects against apoptosis in developing embryos *Hipk1* and *Hipk2* are redundantly required for mouse embryonic survival; *Hipk2* controls neuronal survival and muscular development; *Hipk3* regulates insulin secretion and glucose tolerance; *Hipk4* is required for fertility [83, 161, 170, 232, 233, 239, 247, 248, 249, 250, 251, 252, 253, 254, 256, 280, 341]

### SRPK

Whilst SRPKs appear to play an important role in splicing that is likely critical for development, their expression patterns are also indicative of tissue‐specific functions. SRPK1 is ubiquitously expressed, whilst SRPK2 is enriched in the brain and testes, and SRPK3 mRNA expression is largely restricted to skeletal muscle and the tongue [12, 171, 172]. SRPK1 and SRPK2 proteins are thought to be expressed at higher levels in the brain than SRPK3 [93, 171, 172], although SRPK3 appears to be expressed in specific sub‐sets of neurons [93]. SRPK1 and SRPK2 are enriched in the basal ganglia, hippocampus, cerebral cortex, and cerebellum, with high protein expression in neuronal cells within these regions [171, 172], suggesting a neuronal function for these kinases.

#### SRPK1 is required for early embryonic development

SRPK1 is required for embryogenesis in several model systems. In *C. elegans*, RNAi‐mediated knockdown of *spk‐1* (the SRPK1 orthologue) results in embryonic lethality [173]. In *Drosophila*, there are two SRPK isoforms: *Srpk* and the neuronally expressed *Srpk79d*. Loss‐of‐function of *Srpk* results in oocyte lethality following failure of meiotic spindle microtubule assembly [174], whilst null, inactive, or docking groove mutant *Srpk79d* embryos are viable, but with shortened adult life expectancy and impaired motor behaviour [175, 176]. In mice, homozygous *Srpk1* knockout leads to early embryonic lethality by E14.5, where it performs a highly specialised function of Protamine 1 phosphorylation during parental genome reprogramming in early mammalian embryonic development [90, 113]. In contrast, *Srpk2* knockout mice are viable [177] and *Srpk3* knockout mice display a developmental defect in skeletal muscle growth [14]. Therefore, although SRPK1 is required for early mammalian development and there are other isoform‐specific developmental functions, it is likely that multiple, redundant SRPK isoforms are expressed in most developmental contexts.

#### SRPK in neural development and function

Studies in mammalian and *Drosophila* systems have implicated SRPK in the regulation of neural development and function. In mouse embryonic stem cells (mESCs), SRPKs are proposed to control neural gene expression and neural differentiation *via* phosphorylation of the developmental E3 ubiquitin ligase RNF12/RLIM [93, 178]. In mESCs and human induced pluripotent stem cells (hiPSCs), SRPK phosphorylation stimulates RNF12 catalytic activity and anchors RNF12 in the nucleus *via* unknown mechanisms [93]. SRPK thereby promotes ubiquitylation and proteasomal degradation of nuclear RNF12 substrates, including the transcriptional repressor ZFP42/REX1. As a result, the SRPK‐RNF12 axis patterns genetic programmes that control pluripotency and neural differentiation [93, 178]. This suggests that SRPK‐RNF12 signalling may play a central role in regulating neural differentiation during mammalian development, although this should be addressed by neural‐specific SRPK1/2 mouse knockouts.

Further insight into the role of SRPK in neuronal maintenance derives from *Drosophila* studies. Most notably, the identification of protein aggregates in the axons of motor neurons is associated with reduced levels of the neuronally expressed Srpk79d isoform. In the active zone, Srpk79d phosphorylates the presynaptic active zone protein Bruchpilot on at least seven arginine‐rich N‐terminal motifs [175], and knockout of *Srpk79d* leads to accumulation of Bruchpilot agglomerates, loss of controlled Bruchpilot axonal transport, and aberrant active zone growth and development of synaptic connections. This culminates in failure to maintain nervous system integrity, with locomotor defects and reduced lifespan. [175, 176, 179].

Additionally, a presynaptic function of SRPK has been identified in *Drosophila*. Srpk79d and Bruchpilot co‐localise with the presynaptic transmembrane receptor Lrp4 at or adjacent to the active zone. *Lrp4* knockout results in a loss of olfactory attraction behaviour in *Drosophila* through a reduction in excitatory presynapse number. Srpk79d was found to function downstream of Lrp4 by rescuing this phenotype, and although the mechanism remains to be determined, this reveals a further role for SRPK in active zone organisation and the development of synaptic connections [180]. Similarly, mammalian SRPK2 (but not SRPK1/3) appears to control synaptic function and aggregation of the Bruchpilot homologue CAST1/ERC2. Overexpressed SRPK2 co‐localises with synaptic proteins in rat hippocampal neurons, and co‐immunoprecipitates with CAST1/ERC2 from neural cell lines [181]. This is then thought to disrupt self‐aggregating CAST1/ERC2 complexes that are otherwise required for correct organisation of the presynaptic active zone and subsequent neurotransmitter release [181].

Finally, SRPK2 has been implicated in regulating presynaptic homeostatic plasticity in cultured hippocampal and cortical neurons [182], which enables neurons to scale presynaptic vesicle release in response to changes in their environment. SRPK2 was found to be a key component of the active zone, expressed in the soma, axons, and dendrites, where SRPK2 phosphorylates a RAB3 interactor, Regulating synaptic membrane exocytosis protein 1 (RIMS1), to modulate presynaptic homeostatic plasticity and synaptic glutamate release. Furthermore, in a cortical neuron phosphoproteomics dataset, many SRPK2‐dependent phosphorylation events were identified, with notable enrichment in protein components of active zones [182]. This dataset is indicative of potentially novel substrates of SRPK2 in neurons, the functional relevance of which is yet to be explored.

#### Other developmental functions of SRPK

Further insights into the developmental functions of SRPK have been gleaned from *C. elegans*. *In situ* hybridisation identified enrichment of *spk‐1* (SRPK1 orthologue) in germline cells, and analysis of progeny proliferation following RNAi‐mediated depletion of *spk‐1* found embryonic lethality with incomplete penetrance, with a secondary phenotype of sterility identified in surviving larvae [173]. Germline proliferation and/or maintenance of gametogenesis were disrupted without other major morphological changes in the gonads. Similarly, larval ‘soaking’ with *spk‐1* RNAi (to avoid embryonic lethality) led to low germline proliferation and the development of sterile adults [173], suggesting a function for the *C. elegans* SRPK1 orthologue in germ cells.

Enriched expression of SRPK isoforms in mammalian testes suggests that functions in germline development and fertility may be conserved [12, 93, 172]. Replacement of histones by arginine‐rich protamine during spermiogenesis leads to highly condensed chromatin, which must be reversed upon fertilisation to enable zygotic gene expression. SRPK1 was shown to phosphorylate protamine 1, but not other basic proteins in sperm nuclei [183], to initiate protamine replacement with histones, resulting in zygotic genome activation and paternal genome reprogramming [90]. Further support for the function of SRPK1 in germline development comes from research into the downstream substrate E3 ubiquitin ligase RNF12, which, in addition to regulating neural development, also patterns a genetic programme that controls germ cell differentiation [184]. Furthermore, RNF12 is itself required for efficient spermiogenesis and fertility [185]. Interestingly, RNF12 performs other developmental functions, including regulation of gene dosage compensation *via* X‐chromosome inactivation [186, 187], suggesting that SRPKs may be involved in this process by upstream phosphorylation and regulation of RNF12 function, although these possibilities are yet to be addressed.

### CLK

In comparison to SRPK expression patterns, which indicate possible brain, testes, and neuronal tissue specialisation, CLK1‐4 appear to be ubiquitously expressed in humans with low tissue‐specificity and a common localisation to the nucleoplasm [171, 172]. This may be indicative of their generalised function as splicing factor kinases. However, there is indication of tissue enrichment, where CLK1 displays increased mRNA expression in the brain, bone marrow, and ovaries. Similarly, CLK2 and 3 have high mRNA expression in the bone marrow, and for CLK2, the ovary, uterus, and endocrine tissues, with high protein expression in the lung (for CLK2), or testes, breast tissue, and lymph (for CLK3). The highest mRNA expression of CLK4 is in the retina and brain, and from single‐cell RNA sequencing (scRNAseq) data, CLK4 is enriched in germ and neuronal cell clusters [171, 172]. Although CLK developmental functions are mostly unknown, evidence suggests these are key splicing regulators in stem cells and are associated with several neural and neuroectodermal differentiation and cell cycle phenotypes important for embryonic development.

#### CLK functions in stem cells and development

CLK inhibition leads to profound alterations in gene expression (up to 400 genes) in mouse embryonic stem cells as a result of impaired splicing and intron retention [188]. Thus, CLK activity and splicing regulation may contribute to control genes involved in pluripotent stem cell maintenance and differentiation. However, several lines of evidence point towards specific functions in neural differentiation. CLK1 overexpression induces neuronal differentiation in the PC12 cell line [189], which is associated with activation of components of the ERK1/2‐RSK signalling cascade. Consistent with a role for CLK in neural development and differentiation, CLK1 mRNA was found to be increased during retinoic acid‐mediated neuronal/astroglial differentiation of the mouse embryonal carcinoma cell line P19 but decreased during differentiation into cardiac and skeletal muscle [31].

In *Xenopus*, *Clk2* similarly appears to promote neural development in the embryo. Moreover, Clk2 overexpression augments the induction of several neural markers (e.g. Sox2/3), particularly in the anterior and posterior neural tissues [190]. Clk2 overexpression in combination with a dominant negative BMP receptor mRNA or fibroblast growth factor mRNA further promoted neuroectoderm formation *via* inhibition of Smad1/5/8 phosphorylation [190], suggesting that Clk2 may impact neural differentiation by modulating neuro‐inductive BMP signalling.

The *Drosophila* CLK orthologue, Doa, plays a critical role in differentiation and maintenance of differentiated cells, both in the embryo and developing eye [33]. Homozygous knockout of *doa* is embryonic lethal, and heterozygous mutants display disrupted larval segmentation of the nervous system and disordered development of the eye from the neuroectoderm. Of the rare *doa* homozygote knockout larvae that progress to adulthood, retinal photoreceptors rapidly degenerate, indicating *doa* is required for the maintenance of these cells [33]. These data suggest that CLK is a critical regulator of neural patterning, at least in *Drosophila*.

Interestingly, CLK may also control sex determination in *Drosophila* germ cells, where Doa was shown to regulate alternative splicing [191]. Here, SR protein phosphorylation by Doa and interactions with splicing complex components TRA and TRA2 are required for the initiation of female‐specific splicing through interaction with the enhancer protein doublesex, Dsx. Conversely, the absence of SR protein phosphorylation following *doa* mutation leads to ‘default’ male sex determination [191]. In mice, high levels of CLK3 are detected in mature spermatozoa in the testes [192], suggesting that CLK3 may also regulate aspects of germ cell development and/or function in mammals.

In mouse models, individual CLKs are not required for embryonic survival and are not primarily associated with neurodevelopmental regulation, indicating differences in function and possible redundancy compared to lower organism models. However, phenotypes have been observed upon systematic or tissue‐specific knockout of specific CLK family members. For example, knockout of *Clk1* reduces viral mRNA processing and replication through a reduction in phosphorylation of SRSF3 and altered viral transcript splicing [193]. *Clk2* total and adipose‐specific knockout models show impaired regulation of fatty acid metabolism in brown adipose tissue through reduced CREB phosphorylation and expression of mitochondrial uncoupling protein 1 [194]. Finally, *Clk3* knockout phenotypes are yet to be reported, whilst cardiac‐specific *Clk4* knockout induces the development of cardiac hypertrophy through loss of phosphorylation of the substrate Nexin1 [195].

### DYRK

DYRK kinases have clear developmental functions, with multiple phenotypes observed across organisms. Phenotypic variation between isoform‐specific DYRK knockouts indicates that these kinases perform related roles during development but are not fully functionally redundant. Whilst human DYRK isoforms are for the most part ubiquitously expressed, they can be differentially enriched in a tissue‐specific manner [171, 172]. For example, DYRK1A mRNA is enriched in skeletal muscle and bone marrow, with increased protein levels in the gastrointestinal tract, breast, cervix, and appendix. In the brain, DYRK1A protein levels are highest in the cerebellum, with predominately nucleoplasmic localisation and some synaptic expression in neurons, suggesting possible roles for DYRK1A in transcriptional regulation and trafficking/synaptic function [196]. Indeed, murine DYRK1A is expressed in the adult central nervous system and localised to the nucleus in neuronal cells and during Purkinje cell differentiation [197]. In contrast, DYRK1B exhibits highest expression in skeletal muscle and testis and is found by scRNAseq to be enriched in spermatids, cardiomyocytes, and skeletal myocytes [171, 172]. DYRK2 has low tissue specificity but elevated mRNA levels in skeletal muscle, lymph node, thymus, and the gastrointestinal tract. scRNAseq data also suggests that DYRK2 is specifically expressed by distal enterocytes and retinal horizontal neurons, although DYRK2 is not enriched in the brain in comparison to other organs [171, 172]. DYRK3 and DYRK4 both appear to have low expression levels in most tissues, but with strong mRNA enrichment in the testis [120, 198].

#### Developmental functions of DYRKs

Homozygous *Dyrk1a* ^−/−^ mouse knockout is embryonic lethal, exhibiting severe developmental delay with mid‐gestation death by E13.5. *Dyrk1b* ^−/−^ mice display delayed pre‐weaning lethality by 3–4 weeks [177], although in another study they survive as long as wildtype littermates and are fertile [199]. Consistent with these differing phenotypes, DYRK1A/B appear to act in distinct molecular processes during development. DYRK1A controls a negative feedback loop with the NFATc1 transcription factor, leading to partially blocked osteoclast differentiation in mice [200], whereas DYRK1B contributes to the regulation of myoblast differentiation by phosphorylating p27 and Cyclin D1, and HDAC5/9 in murine myoblasts [128, 133]. This leads to modulation of protein stability and de‐repression of myocyte enhancer factor 2 (MEF2C) by interfering with the nuclear accumulation of inhibitory HDACs through phosphorylation of the HDAC NLS [201]. In mouse myoblasts, DYRK1B also phosphorylates p21 at S153 to localise p21 in the cytoplasm, thereby reducing cell cycle inhibition by p21 and suppressing apoptosis [129]. Interestingly, in zebrafish, dyrk1b promotes skeletal muscle differentiation, stabilisation of 4e‐bp1 activity, and induction of autophagic flux, and the *dyrk1b* knockout exhibits embryonic lethality [202].

DYRK2 also performs essential developmental functions. In *C. elegans*, knockout of the DYRK2‐3 orthologue *mbk‐2* exhibits spindle positioning defects during cytokinesis and resulting embryonic lethality [124]. MBK‐2, alongside other key proteins (such as CDK‐1 and GSK‐3), is also required for oocyte‐embryo transition by marking the RNA‐binding protein OMA‐1 for ubiquitin‐mediated degradation. Thus, inactive mutants of MBK‐2 stabilise OMA‐1 after meiosis, leading to mitotic failure and embryogenesis [72, 122, 123]. *Dyrk2* ^−/−^ mice are viable but are characterised by reduced Hedgehog (Hh) signalling, resulting in abnormal ciliary morphology, genesis, and skeletal development [203]. Similarly, there are multiple malformations in these mice with craniofacial, limb, and organ developmental defects, resulting in the failure of pups to survive after birth [204]. Indeed, other DYRKs have established positive and negative regulation of Hh pathway activity, which is crucial for embryogenesis and controlled development, reviewed in Ref. [205].

*Dyrk3* and *Dyrk4* murine knockouts are viable and fertile [198], although some observations point towards developmental functions. DYRK3 has been shown to be a regulator of erythropoiesis and a survival factor for haematopoietic progenitor cells [206], possibly *via* regulation of CREB S133 phosphorylation, interaction with Protein Kinase A, and attenuation of Interleukin 3‐induced apoptosis. DYRK3 is further suggested to selectively attenuate erythropoiesis during anaemia by inhibiting NFAT transcriptional response pathways [207]. Specific DYRK4 developmental functions have not yet been reported; despite high testes expression, DYRK4 is not required for testes development or spermatogenesis and does not impact the morphology or mobility of spermatids [198]. This suggests that the developmental roles of DYRK4 may be masked by functional redundancy with other DYRKs.

#### DYRK phenotypes relating to neurological development and functioning

A major function of DYRKs is co‐ordination of neural differentiation and function. Diverse metazoan DYRK1A genetic models present related neurodevelopmental abnormalities, suggesting there is functional conservation in brain development [208, 209, 210]. *Drosophila mnb* knockout animals display abnormal spacing of neuroblasts and incorrect brain development with reduced size, leading to behavioural abnormalities in learning, memory, vision, and olfactory tasks [59]. Interestingly, in *C. elegans*, whilst the loss‐of‐function mutant of *mbk‐1* (DYRK1A‐B homologue) has no major defects of morphology or locomotion, increased *mbk‐1* gene dosage leads to a defect in olfactory sensing [124], paralleling gene dosage effects on neurodevelopmental function observed with mammalian DYRK1A.

#### DYRK1A is a major regulator of neurodevelopment and function

Several lines of evidence suggest a key role for DYRK1A in neural development. *Dyrk1a* is highly expressed in the mouse neural tube, and *Dyrk1a* ^−/−^ knockout leads to embryonic lethality by E14.5 with severe developmental delay [208]. Gene dosage is critical, as heterozygous *Dyrk1a* ^+/−^ mice are viable but have microcephaly, reduced body weight in adults, and motor deficiencies [208, 211]. *Dyrk1a* ^+/−^ animals also display pre‐weaning neurological phenotypes, such as delayed eyelid and ear opening and specific neurological behaviours controlling freezing and startle response, although neuromuscular function is unaffected [208]. Further evidence supporting an important function for DYRK1A in the development of the central nervous system is discussed later *via* the role of DYRK1A in Trisomy 21/Down's syndrome [212, 213].

DYRK1A may also function in the regulation of neural progenitors and stem cells. In neural stem cells, DYRK1A acts downstream of EGFR signalling to co‐ordinate asymmetrical neural stem cell division in a gene dosage‐dependent manner, whereby heterozygous DYRK1A expression impairs neural stem cell self‐renewal in response to EGF stimulation [214]. In this context, the sub‐cellular distribution of DYRK1A affects the symmetry of stem cell division, and DYRK1A was found to antagonise EGFR endocytosis to ‘brake’ EGF signalling [214]. DYRKs can also facilitate neuronal differentiation by interacting with the RAS/B‐RAF/MEK signalling pathway in neuronal cells, although interestingly, this effect may be independent of DYRK catalytic activity [142]. Further evidence towards a role for DYRK1A in neural progenitor regulation is found in a human embryonic stem cell (hESC) model of neural specification upon dual‐SMAD inhibition, where both chemical and genetic approaches inhibiting DYRK1A blocked the differentiation of hESCs into the neural lineage [215]. Expression levels of the early human neural progenitor marker PAX6 were found to mirror the expression levels of induced or inhibited DYRK1A during neural progenitor formation [215]. DYRK1A therefore has an important role in regulating neural differentiation from stem cells; however, the precise mechanism by which DYRK1A controls the response to differentiation induction signals remains to be established.

Correct DYRK1A gene dosage also appears to be critical for neuronal development. In DYRK1A transgenic mice (with ~ 1.5 fold increase in DYRK1A expression), there is decreased neuronal cell death with impaired proliferation of embryonic cortical neuronal precursors, which corresponds with increased p53 S15 phosphorylation and p21(cip1) accumulation [131]. DYRK1A may also block HIP1‐mediated cell death during differentiation of hippocampal neuroprogenitors by phosphorylating HIP1 [216]. DYRK1A overexpression can also lead to premature neural development and reduced progenitor proliferative capacity. *In utero* electroporation of DYRK1A into the neonatal cortex drives nuclear speckle‐like localisation of DYRK1A in mouse embryonic brain slices, which may suggest a function in co‐localising with splicing factor proteins in a manner reminiscent of SRPK/CLK [115]. DYRK1A overexpression also reduces neural progenitor proliferation and promotes premature neural development and translocation through the murine neocortex [217]. Similarly, DYRK1A overexpression prevents proliferation of cultured mouse neuroblasts by increasing cyclin D1 nuclear export and aggregation of cyclin D1 [217]. This could be due to DYRK1A control of the transcriptional regulatory REST complex, which is expressed in neural progenitors to drive proliferation and repress neural differentiation. In this context, DYRK1A overexpression leads to ubiquitin‐mediated degradation of the REST complex [218, 219, 220]. In summary, DYRK1A expression must be exquisitely controlled, as either subtle loss or gain of expression and/or function may lead to neurodevelopmental abnormalities.

#### Neurological functions of other DYRK kinases

Several other DYRK family members have described neurological functions, and the role of DYRK2 in neuronal development has been recently reviewed elsewhere [221]. In zebrafish, *dyrk1b* knockout is embryonic lethal due to a failure to develop myotomes from single spinal nerve roots [202]. Heterozygous *Dyrk1b* ^−/+^ mice have decreased startle response and increased pre‐pulse inhibition, indicative of altered neurological development and behaviour [222]. The expression of *Drosophila* Dyrk2 (orthologue of human DYRK4) increases during larval development, and *dyrk2* ^−/−^ flies display a reduced smell‐avoidance phenotype [223], suggestive of neuroectodermal abnormalities. *Dyrk2* mRNAs are present in the morphogenetic furrow and eye‐antennal discs, but apart from a reduced photoreceptor depolarisation response, homozygous *dyrk2* ^−/−^ flies otherwise exhibit no overt changes in eye function or morphology. Interestingly, however, *dyrk2* overexpression leads to a ‘rough eye’ patterning defect with increased bristles, and smaller eye size [223], suggesting that DYRKs other than DYRK1A may have neuroectodermal functions for which gene dosage is critical. In support of this notion, overexpression of DYRK isoforms in rat hippocampal neuronal cultures was found to alter neuronal outgrowth and axon branching in isoform‐specific manners [224]. Overexpression of catalytically active Dyrk3 and Dyrk4 increased dendrite branching, whereas Dyrk1A reduced axon length, and Dyrk2 shortened both axon and dendrite length and branching. This is suggested to occur *via* phosphorylation of the cytoskeletal component doublecortin, reducing doublecortin enrichment at neurite tips and altering neurite morphology [224]. Other studies have also explored the role of DYRK2 in phosphorylating proteins influencing neural growth cone dynamics and morphogenesis, for example, Collapsin, Collapsin response mediator proteins (CRMPs), and Nuclear distribution element‐like 1 (NDEL1) [225, 226]. As correct and balanced neuronal morphogenesis and growth are critical for proper neuronal function and brain development, it is unsurprising to find that myriad alterations in DYRK expression and function underlie neurodevelopmental disorders, which will be discussed later.

#### DYRKs in ciliogenesis

DYRK kinases have been found to regulate ciliogenesis, which plays a key role in various developmental contexts, including neurological development and function. In *Xenopus* multi‐ciliated cells, Dyrk1a interacts with and phosphorylates the centrosomal protein Cep27 to enhance recruitment of Polo‐like kinase 1 (Plk1). This promotes mature centriole disengagement required for the migration and docking of basal bodies to the apical surface for multi‐ciliated cell maturation. Knockdown of *dyrk1a* and *cep27* reduces levels of acetylated tubulin in cilia and leads to defects in multi‐ciliated cells through failure to disengage mature centrioles [227]. In *Xenopus* embryos, *dyrk1a* is strongly expressed in the brain, spinal cord, optic vesicles, the developing heart, and in ciliated cells [228]. Depletion of *dyrk1a* leads to failed ciliogenesis and reduced brain size, which are thought to occur as a result of Dyrk1a localising to and regulating mitotic spindle dynamics [228]. Interestingly, this finding echoes Dyrk1a‐regulated microtubule dynamics during *Drosophila* dendrite development, where Dyrk1a phosphorylation of β‐tubulin inhibits polymerisation [229]. Human DYRK2 also associates with the EDVP E3 ligase complex implicated in centriole length and cilia assembly [143, 144]. In this complex, DYRK2 phosphorylates centrosomal protein CP110, leading to its ubiquitylation by other complex members and suppression of cilia assembly [143]. Moreover, DYKR2 is required for Hedgehog (Hh)‐mediated signalling in early mouse development, where *Dyrk2* ^−/−^ embryos present with skeletal defects and elongated cilia with abnormal morphology, contributing to impaired trafficking of Hh signalling components GLI1 and PTCH, with resulting impact on tissue development [203, 204]. Therefore, alterations in *DYRK1A* levels may impact the cilia function required for correct early embryonic development and proper neurological function.

### HIPK

In comparison to their closest relatives, DYRKs, human HIPKs are expressed in a highly tissue‐specific manner. HIPK1 mRNA is enriched in the bone marrow, thymus, testis, retina, and cerebellum, with scRNAseq identifying enrichment in ciliated cells and spermatids [171, 172]. Analysis of mRNA expression patterns finds HIPK2 predominately expressed in the brain, central nervous system, kidney, and lymph [230]. Brain HIPK2 expression is not appreciably region‐specific but exhibits cell‐type‐specific enrichment in oligodendrocytes [171, 172]. HIPK3 exhibits strong mRNA expression in the tongue, skeletal muscle, and brain; single‐cell analyses suggest HIPK3 is expressed mostly in adipocytes, endothelial cells, and cardiomyocytes. Finally, HIPK4 has highly restricted tissue expression, limited to the testis and brain [171, 172]. Although the HIPK4 primary sequence and structure diverge from HIPK1‐3, HIPK4 expression overlaps with other family members, which may lead to functional redundancy in key developmental roles.

#### Emerging functions of HIPKs in development

In mammalian development, HIPK2 can regulate myoblast differentiation, where HIPK2 complexes with histone deacetylases HDAC3 and HDAC4, translocating to the nucleus, and phosphorylating the transcription factor myocyte enhancer factor 2 (MEF2C) [231]. Additionally, this complex of HIPK2 is required for HDAC3‐dependent deacetylation of MEF2C [231]. Together, these repress MEF2‐dependent gene expression in undifferentiated myoblasts. This is in contrast to the role of DYRK1B in the regulation of myoblast differentiation, which interferes with the nuclear accumulation of inhibitory HDACs leading to the de‐repression of the *Mef2c* gene [128]. Interestingly, HIPK2 itself is regulated by caspase cleavage that increases during differentiation, thereby relieving inhibition of MEF2C and enabling myoblast differentiation [231].

Another interesting function of HIPK kinases appears to be in the regulation of fertility. Firstly, in *C. elegans*, the kinase domain of the HIPK1 orthologue, HPK‐1, is required for germline proliferation and therefore normal brood size [232]. Secondly, in mice, HIPK4 displays a highly specific developmental phenotype; it is highly expressed in the testis, and knockout results in defective spermatogenesis and male sterility [233]. *Hipk4* ^−/−^ animals exhibit reduced sperm motility, abnormal head morphology, DNA fragmentation, and diminished oocyte binding. Heterozygous *Hipk4* ^+/−^ mice have similarly reduced sperm motility but normal morphology [233]. *Hipk4*‐deficient mouse testes exhibit few transcriptional changes relative to wildtype, but proteomic analysis of cultured mouse embryonic fibroblasts expressing wildtype or kinase‐inactive HIPK4 found noticeable differences in multiple actin regulators [233]. Dysregulation of F‐actin and actin capping proteins in spermatids leads to the failure of correct elongation and differentiation of spermatids upon mechanical pressure [233]. The precise mechanism of how HIPK4 regulates cytoskeletal proteome dynamics is not known but does not appear to be *via* direct transcriptional regulation. Since mammalian HIPK4 is divergent from HIPK1‐3, further research is required to explore whether other HIPK isoforms (such as HIPK1 which has enriched mRNA levels in spermatids) can similarly regulate fertility and spermatogenesis.

HIPKs have been more widely implicated in cell fate determination. HIPK4 was identified from RNAi screening as a negative regulator of human induced pluripotent stem cell differentiation into skin epithelium in a kinase activity‐dependent manner [234]. Additionally, HIPK2 can localise to chromatin and function as a coactivator of gene transcription [167]. Interestingly, HIPK2 SUMO binding capability rather than kinase activity is required for the de‐condensation of chromatin [235]. SUMOylation can act as a dynamic labelling mark during mitosis and as a positive or negative regulator of gene transcription [236, 237]. Differing levels of SUMOylation can also facilitate cell‐fate transitions, such as the direct transdifferentiation of mouse embryonic fibroblasts into mature neurons and reprogramming into induced pluripotent stem cells [238], which positions HIPKs as potential regulators of developmental processes.

#### HIPK regulation of developmental signalling

HIPK2 can modulate several developmental signalling pathways, for example Notch [239], Hippo [240, 241], Wnt [242], Hedgehog (Hh) [243], and JAK–STAT [244]. Co‐ordination of such pathways is critical for proper development, and when dysregulated, these pathways contribute to developmental disorders, or tumorigenesis, and metastasis [245]. For example, whilst the loss of Drosophila *Hipk* is embryonic lethal, RNAi reduction of *Hipk* leads to defects in wing development similar to those of impaired Wg/Wnt signalling, and ectopic expression of *Hipk* rescues phenotypes of Wnt loss [242]. Moreover, *Hipk* can negatively regulate an E3 ubiquitin ligase complex to suppress the degradation of Wnt/Hh signalling effectors β‐catenin(Arm) and Gli1(Ci) and promote the transcriptional activity of these developmental pathways [242, 243]. HIPK2 regulation of Wnt and Hh pathways parallels functions seen for DYRKs, particularly DYRK2, in early development and may serve as a point of convergence between morphogen signalling and tissue size and development with cell density and mechanical stress or tension. Indeed, *Drosophila* Hipk regulates Yorkie (homologue of YAP) nuclear localisation as part of the Salvador‐Warts‐Hippo (SWH) pathway to regulate tissue growth, although YAP is unlikely to be a direct target of HIPK2 in humans [246]. The regulation of Yorkie in *Drosophila* affects its localisation, whereas the regulation of human YAP may affect its protein levels.

#### HIPK functions in the nervous system

HIPKs also have reported roles in the central nervous system and in musculoskeletal structure and function. Notably, HIPK1 and 2 function redundantly during development, where *Hipk1/2* double knockout mice, but not individual knockouts, are embryonic lethal following failure in neural tube formation, defective angiogenesis, and haematopoiesis [247, 248, 249]. HIPK2 co‐ordinates the survival of sensory, sympathetic, and midbrain dopamine neurons, and therefore *Hipk2* deletion leads to neuronal loss, a myopathic or cardiac phenotype, and altered morphology of neurons and neuromuscular junctions, echoing phenotypes observed in *Drosophila* [250, 251, 252, 253, 254]. Interestingly, *Drosophila Hipk* displays dosage sensitivity, with both knockout and overexpression leading to lethality [255]. Notably, Hipk regulates neuromuscular junction size by altering the number and size of muscle nuclei [255]. Hipk can also synergise with Notch signalling for eye patterning and development in *Drosophila* through the repression of Groucho. Conversely, inactivation of Hipk results in a small eye phenotype with loss of photoreceptors [169, 239]. Additionally, *Hipk2* ^−/−^ mice show reduced activation of JNK‐c‐JUN signalling and a resultant failure to maintain the ratio of ionotropic glutamate receptors GluN2A, 2B, and 2C during neuronal development. Subsequent changes in levels of these receptors drive increases in ERK1/2 signalling, CREB phosphorylation, and synaptic activity genes, thereby promoting neuronal survival [253]. Finally, HIPK2 has been implicated as a cofactor for TGFβ/BMP signalling *via* interaction with regulatory (R)‐SMAD transcription factors to promote pro‐survival signalling. This occurs particularly in enteric dopaminergic neurons, where the loss of HIPK2 or TGFβ3 (but not TGFβ1) results in dopaminergic neuron deficiencies and an increase in phospho‐SMADs in enteric neurons. Increased apoptosis in these cells results in psychomotor abnormalities [252, 254].

Recent advances have also been made in unpicking the developmental redundancy of human HIPKs by reconstituting human HIPKs into a *Drosophila Hipk* ^−/−^ model to assess survival, morphology, and limb development phenotypes [256]. Confirming findings that murine HIPK1/2 redundantly mediate sonic hedgehog (Shh)‐induced proliferation of the mesoderm and neural tube [249], both human HIPK1 and HIPK2 rescue the lethality of *Hipk* knockout in flies. In contrast, human HIPK3/4 are more divergent in function and are unable to rescue this phenotype. Interestingly, the same *Drosophila Hipk* ^−/−^ model identified distinct functions of human HIPK1 and 2 in the rescue of head defects; whilst both rescued lethality, only human HIPK2 facilitated correct eye, ocellar, and bristle development from the neuroectoderm [256]. Therefore, although more research is needed, initial evidence suggests there are specific functions of HIPK sub‐family members in neurodevelopment.

### Summary

There are many established and emerging cellular mechanisms (Table 2) and genetic phenotypes (Table 3) that are associated with the related SRPK, CLK, and DYRK kinase families during development. A wide range of *in vitro* and *in vivo* studies highlight roles for these kinases in cellular signalling pathways, tissue development, stem cell differentiation, and correct neuronal activity. Defects in critical pathways during early development lead to embryonic lethality, as observed in multiple model organism studies. Importantly, whilst kinase isoforms exhibit functional overlap, the families are typically not entirely redundant during development. Notably, genetic ablation of SRPK1, CLK1, DYRK1A/B/2, and HIPK1&2 orthologues results in embryonic lethality in both lower and higher organisms. Interestingly, however, in mice, the additional isoforms SRPK2/3, CLK1‐4, DYRK3/4, and HIPK3/4 are not embryonic lethal, indicating only partial functional redundancies within the kinase families.

The expression patterns of the SRPK, CLK and DYRK kinase families are similar, with most isoforms ubiquitously expressed. However, apparent tissue‐specific enrichments are suggestive of specialised functions. Several SRPK, DYRK, and HIPK isoforms display enrichment in the brain, skeletal muscle, bone marrow, and testes, which may underlie phenotypes observed in model organisms such as defects in neuroectodermal and musculoskeletal formation and spermatogenesis. Interestingly, CLK isoforms exhibit somewhat less tissue‐specific expression than SRPK isoforms, which may reflect a greater generalised function for splicing control during development. However, kinase expression in differentiated adult cells is unlikely to accurately reflect developmental expression dynamics, and so comparisons to genetic phenotypes may be misleading.

There are further commonalities between HIPK, DYRK, and to a lesser extent CLK in regulating developmental signalling pathways such as Hh, Wnt, and TGFβ/BMP, which impact tissue development including ciliogenesis, mesodermal development, and neuroectoderm formation. However, the precise mechanisms underlying these genetic phenotypes remain to be elucidated. Additionally, SRPK/CLK/DYRK families control the capacity of stem cells and progenitors to differentiate into and properly function as varied cell lineages, such as neural lineages, osteoclasts (DYRK), myoblasts (HIPK), and gametes (SRPK/CLK/HIPK). For example, SRPK may regulate stem cell pluripotency and neural differentiation, whilst CLK has similarly been suggested to regulate neural patterning. SRPK and HIPK are associated with proper sensory and motor function through the correct development of synaptic connections, neurotransmitter release, and neuronal cell survival signalling. DYRK kinases, especially DYRK1A, are critical regulators of development including neurodevelopment and function, whereby an imbalance in gene dosage can profoundly affect signalling within stem cells and the embryo, resulting in significant developmental consequences, including regulation of neuronal stem cell self‐renewal, asymmetric cell division, differentiation of neural progenitors, neural morphogenesis and growth cone dynamics.

## Dysregulation of SRPK, CLK, and DYRK kinases in developmental disorders

**Fig. 3:**
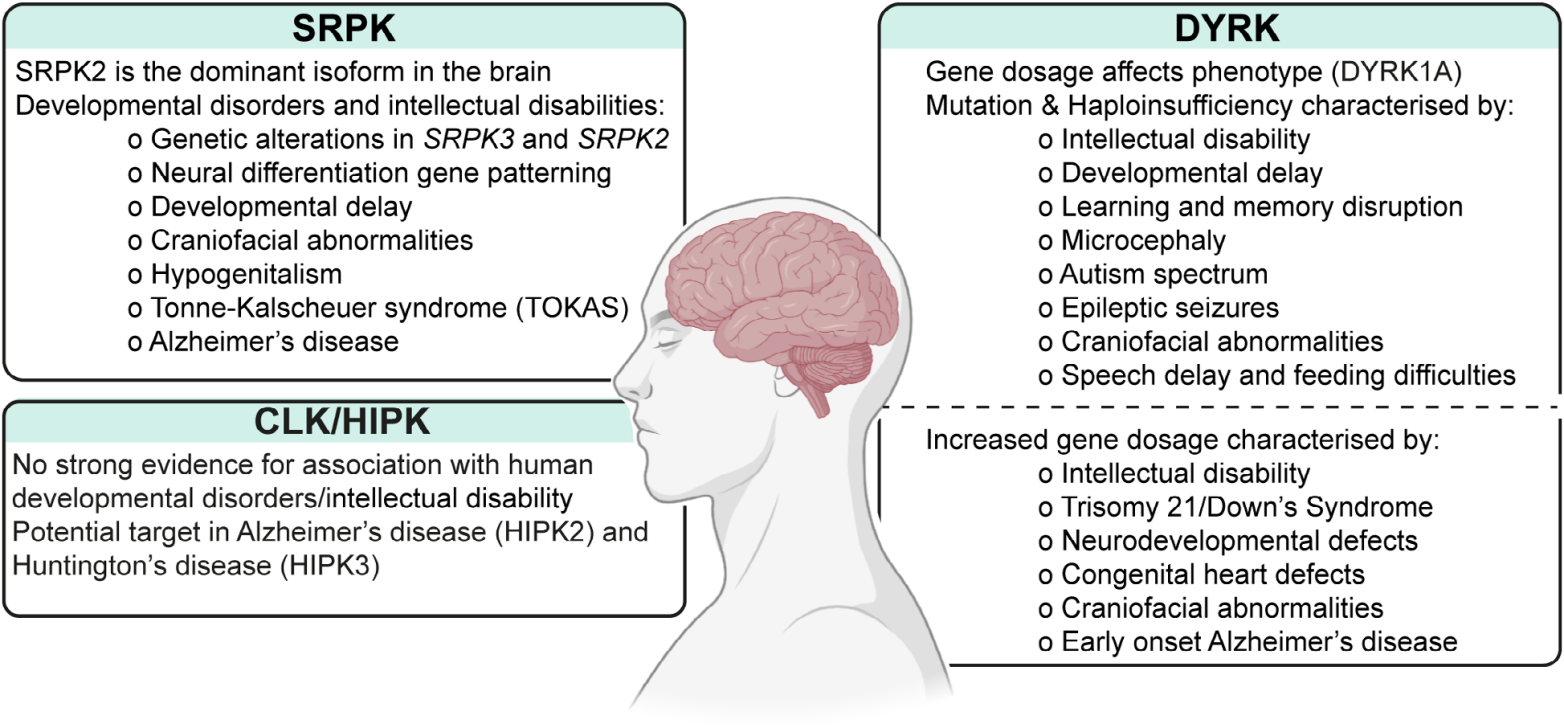
Involvement of SRPK, CLK, and DYRK kinase families in neurodevelopmental and neurological disorders. Created with BioRender.com.

As a result of the key roles played by CMGC kinases in developmental regulation, it is thus important to highlight the human diseases and disorders arising from their dysregulation. We now discuss evidence that the functions of SRPK and related kinases are disrupted in developmental disorders, in particular their association with neurological disorders (Fig. 3).

### SRPK

SRPKs play key roles across the developmental continuum. However, as discussed earlier, SRPKs are highly expressed in the brain, and much of the developmental data suggests that SRPKs play key roles in the development of the nervous system. Therefore, several reports have identified pathological functions for SRPK‐mediated signalling in neurodevelopmental disorders such as intellectual disability.

#### An SRPK signalling pathway disrupted in intellectual disability

SRPK kinase activity regulates a neurodevelopmental pathway *via* phosphorylation of the E3 ubiquitin ligase RNF12/RLIM [93, 178]. The importance of this pathway for the development of the nervous system is underscored by the finding that RNF12 is mutated in patients with a syndromic form of X‐linked intellectual disability recently termed Tonne‐Kalscheuer syndrome (TOKAS; [257, 258, 259, 260]). TOKAS is characterised by impaired adaptive and cognitive functions, craniofacial abnormalities characteristic of intellectual disability, as well as syndromic features including diaphragmatic hernia, urogenital abnormalities, and velopharyngeal insufficiency [257, 258, 259]. RNF12 patient variants largely disrupt catalytic E3 ubiquitin ligase activity [178, 257], which leads to impaired signalling *via* the SRPK‐RNF12 pathway. This results in failure to efficiently ubiquitylate nuclear RNF12 substrates, including the YY1‐family transcription factor ZFP42/REX1. Expression of neurodevelopmental genes is thereby dysregulated, which may underpin intellectual disability features characteristic of TOKAS patients [178].

Consistent with its function in the RNF12 pathway, SRPK family gene alterations are also associated with intellectual disability. Several deletions have been reported in *SRPK2* [93, 261, 262], which is thought to be the major SRPK isoform in the brain. Furthermore, a number of missense variants have been reported in *SRPK3* [258], which is expressed in a specific sub‐set of neurons [93]. These variants, in some cases, disrupt *SRPK3* phosphorylation of RNF12, which is predicted to lead to impaired signalling. It is not yet known where and when SRPK‐RNF12 pathway activity is required for neurodevelopment, or how dysregulation of signalling leads to intellectual disability. Interestingly, the discovery of *SRPK2* heterozygous deletions and *SRPK3* amplifications associated with intellectual disability [93, 261, 262] suggests that gene dosage is critical for correct regulation of SRPK signalling. In support of this notion, RNF12/RLIM amplifications, in addition to deleterious variants, have been associated with neurological phenotypes and facial features characteristic of impaired developmental and intellectual disability [263]. These findings highlight that signalling *via* SRPKs is finely tuned to regulate healthy embryonic and, more specifically, neuronal development, and echo the dosage‐sensitive nature of DYRK1A required for correct neurological development.

### DYRK

Similar to SRPKs, evidence for critical developmental functions of DYRKs is connected to neural expression and neurodevelopmental phenotypes. A variety of DYRK‐dependent mechanisms identify both isoform‐specific, and pan‐isoform control of aspects of proliferation, ciliogenesis, cell cycle, ubiquitin‐mediated degradation, and neuroectoderm development. These functions are closely linked to various pathological phenotypes, with which DYRKs (predominately DYRK1A) have been associated.

#### DYRK1A is a major player in neurodevelopmental disorders

DYRK1A is heavily implicated in neurodevelopmental disorders, with alterations in the *DYRK1A* gene frequently associated with intellectual disability. As discussed earlier, proper development is sensitive to DYRK1A dosage, and both increased and reduced expression of DYRK1A can lead to neurodevelopmental anomalies [264]. Increased DYRK1A levels resulting from gene duplication, such as in Trisomy 21 or Down's Syndrome, are characterised by neurogenic and neurodevelopmental defects, congenital heart defects, leukaemia, and early onset neurodegenerative disease. Reduced *DYRK1A* expression, truncations, or deletions, such as in DYRK1A haploinsufficiency syndrome/DYRK1A‐related intellectual disability syndrome, are characterised by intellectual disability, developmental and learning disruption and delay, microcephaly, autism spectrum, epileptic seizures, speech delay, and feeding difficulties [209, 265, 266, 267]. This may arise as a result of an imbalance in excitatory and inhibitory signalling in neurons that promote GABA production and subsequent reduced GABAergic neurotransmission [268]. Importantly, altered DYRK1A protein levels appear to be causative based on murine models, as artificially reducing DYRK1A expression is sufficient to rescue major aspects of motor impairment and hypoactive behaviour in transgenic DYRK1A overexpression animals [269]. Although most research has focussed on DYRK1A, there is also a recent report of DYRK1B haploinsufficiency in a family with mild and severe intellectual disability, seizures, autism, obesity, and other symptoms [270]. Additionally, a transcriptomic and functional enrichment analysis of resected tissue from a subset of patients with epilepsy has associated downregulation of DYRK2 with an epileptic and neuronal apoptotic gene network [271]. Together, these suggest that other DYRK family members may have neurological functions that are disrupted by disease.

#### DYRK1A is encoded on chromosome 21 – implication in trisomy 21/Down's syndrome

The *DYRK1A* gene is located at 21q22.2, which has been identified as the Down's syndrome critical region (DSCR) of Chromosome 21. Thus, DYRK1A has long been considered a key player that potentially underlies the characteristic presentations of Trisomy 21, with the third copy of the *DYRK1A* gene implicated in disease aetiology. In support of this hypothesis, overexpression of DYRK1A in transgenic mice leads to behavioural and motor abnormalities (e.g. memory and spatial learning), suggesting that DYRK1A protein levels are critical for correct neurological development and/or function. Furthermore, in a mouse model of Trisomy 21 (segmental trisomy 16/Ts65Dn mice), overexpression of *Dyrk1a* by 1.5‐fold echoes phenotypes of Trisomy 21 observed in humans [131, 197, 212, 213, 269, 272, 273], thus supporting the notion that elevated DYRK1A may underpin neurodevelopmental abnormalities associated with Trisomy 21.

Several mechanisms for DYRK1A function in Trisomy 21 have been proposed. For example, DYRK1A can phosphorylate Notch to attenuate its signalling, thereby antagonising Notch‐dependent inhibition of neuronal differentiation [274]. DYRK1A may also promote neuronal differentiation by inducing G0/G1 arrest *via* phosphorylation of Cyclin D1 and p27(kip1) [127], and regulate neuronal maturation by promoting ubiquitin‐mediated degradation of the REST transcriptional regulator [210, 218, 220, 275]. Thus, increased DYRK1A kinase activity in Trisomy 21 patients has the potential to drive neurological phenotypes associated with Trisomy 21. Therefore, DYRK1A inhibition may present a therapeutic intervention for Trisomy 21, with the potential to manage and/or reverse adverse neurological symptoms, including the increased risk of neurofibrillary degeneration and the development of dementia [116, 276, 277].

### CLK

There are few direct reports of CLK regulation and function in relation to human developmental disorders, although developmental phenotypes arising from model organism studies suggest CLKs have a role in neurodevelopment *via* splicing regulation. Notably, heterozygous knockouts of *Drosophila* CLK (*Doa*) display distinct neurological abnormalities, in contrast to *Doa* ^−/−^ embryonic lethality [33]. CLK3 is highly homologous to Doa and is similarly enriched in gametes, suggesting that CLK3 may be the functional orthologue of Doa in early mammalian development. Furthermore, and as discussed previously, CLK2 promotes neural tissue formation in *Xenopus* by regulating the BMP and FGF signalling pathways that are crucial for development [190]. Further key developmental functions are suggested by altered expression of ~300–400 genes following CLK inhibition in mouse embryonic stem cells, which occurs *via* impacts of CLK inhibition on splicing activity such as intron retention [188].

From the DECIPHER database of developmental disorders, *CLK1‐4* gene alterations are typically found within large chromosomal areas of deletion or duplication, and the few identified single nucleotide variants (SNVs) within the gene family are of unspecified clinical significance. However, there may be rare associations between CLK variants and developmental abnormalities. One *CLK2* loss‐of‐function variant was identified in two developmental disorder patients with abnormalities of the head, neck, eye, endocrine system, and nervous system [261, 262]. Similarly, one *CLK3* in‐frame deletion was identified in a patient with abnormalities in the head, neck, eye, musculoskeletal system, and nervous system [261, 262]. The direct clinical relevance of these variants has yet to be demonstrated, and there are currently no strong links between CLKs and human developmental disorders. However, together with available experimental evidence, CLKs likely have key developmental functions that are performed redundantly between family members, warranting further investigation of their relevance and potential disruption in human developmental disorders.

### HIPK

As with CLKs, there is currently little information about the HIPK sub‐family of protein kinases in human developmental disorders. Most information pertains to model organisms and the contexts of DNA damage and ER stress responses. However, there are reported functions of HIPKs in cancer (HIPK regulates multiple growth and developmental pathways [152, 244, 245, 278]), proteinopathies, and motor neuron disease (e.g., Amyotrophic Lateral Sclerosis through HIPK‐mediated ER‐stress response‐induced neurodegeneration [279] and regulation of neuromuscular junctions [255]), and eye development, where in model organisms HIPK loss affects ectoderm and neuroectoderm formation. In *Drosophila*, *Hipk* gene knockout is lethal, with multiple abnormalities including failed ocellar development and loss of photoreceptors, and whilst human HIPK1 and 2 transgenes could rescue this phenotype, HIPK3 and 4 could not, indicative of unique functions of distinct HIPKs [239, 256, 280]. This is echoed by individual murine *Hipk1‐4* knockouts, which present distinct phenotypes of neuronal loss, myopathy, impaired glucose tolerance, and infertility [161, 233, 250, 254].

As is the case for *CLK*, *HIPK* gene alterations have been found within large areas of chromosomal deletion and duplication in patients with various developmental disorders (DECIPHER database), but with no SNVs of verified clinical relevance. Missense mutations have been identified for *HIPK1*, *3*, and *4*, although it is unclear how these variants contribute to pathogenic phenotypes, in contrast to the multiple loss‐of‐function variants predicted for SRPK2‐3. However, patients with HIPK gene variants exhibit phenotypes of abnormal development or function of the endocrine system, nervous system, eye, and ear, and/or global developmental delay, indicative of a potential function in developing ectodermal tissues that reflect observations in model organisms [261, 262].

## Therapeutic potential of SRPK, CLK and DYRK kinase families in developmental disorders

### Current approaches to exploiting SRPK, CLK and DYRK kinase families for therapy

SRPK, CLK, DYRK, and sub‐family HIPK have been associated with several disease indications, with potential therapeutic strategies either discussed or being pursued. Disease areas include viral infection and replication [193, 281, 282, 283, 284], tumour angiogenesis [285, 286], and other aspects of tumour growth and biology [2, 113, 287, 288, 289]. The diverse roles of DYRKs in cancer have been extensively reviewed elsewhere [126, 290, 291, 292, 293]. Aberrant splicing has also been proposed as a ‘hallmark’ of cancer, with splicing inhibition highlighted as a therapeutic strategy [294, 295]. In several cancer types there are high levels of alternative intron retention over that of matched normal tissue [296], indicating splicing inhibitors could be effective in restoring normal function.

For the related SRPK, CLK, and DYRK kinase families, their roles in controlling splicing in cancer development and angiogenic conditions such as diabetic retinopathy have been well described [2]. Most notably, SRPK controls a splicing switch between pro‐ and anti‐angiogenic vascular endothelial growth factor (VEGF) isoforms, where SRPK inhibition blocks angiogenesis [297, 298, 299, 300, 301]. Similarly, SRPK1 can control alternative splicing of MAP2K2 (a component of the MAPK pathway), where inhibition or knockdown of SRPK1 in pancreatic cancer cell lines led to increased sensitivity to chemotherapy agents [302]. Overexpression of SRPK1 in cancer lines may thus contribute to tumorigenic cell signalling behaviour. Indeed, increased SRPK1 expression is observed in several tumour cell lines and found to be a genetic vulnerability in acute myeloid leukaemia [302, 303, 304]; therefore, SRPK1 inhibition is suggested as an effective strategy to reduce tumour growth [305, 306]. Similarly, SRPK2 inhibition and/or dual inhibition of mTOR and O‐linked β‐N‐acetylglucosamine (O‐GlcNAc) in metabolic syndromes or cancer cells dependent on upregulated lipid metabolism has been proposed as a potential anti‐cancer therapy [288, 307]. SRPK2 is associated with the initiation of lipogenic gene pre‐mRNA splicing regulation following activation of the nutrient‐responsive pathways mTORC1‐S6K1 [288] and the hexosamine biosynthetic pathway [307] that promote cell growth. Therefore, inhibition of SRPK2 or dual inhibition of mTOR and O‐GlcNAc could be promising to suppress the growth of cancer cells dependent on upregulated lipid metabolism. However, SRPK1 inhibition or downregulation has also been linked with cis/carboplatin chemotherapy resistance, highlighting that the therapeutic benefit of SRPK inhibition is likely tumour‐type and/or isoform dependent [308, 309, 310].

Interestingly, whilst HIPKs were initially thought of as tumour suppressors by controlling DNA damage response and apoptosis [152], some reports indicate an overexpression and oncogenic function of HIPKs in some cancers [311, 312]. Additionally, the use of CLK inhibitors correlates with an anti‐proliferative effect in cancer lines thought to arise from altered splicing of genes associated with DNA replication, repair, and mitotic processes [44, 313, 314, 315, 316]. Therefore, where inhibition of SRPK/CLK/DYRK kinase families may lead to cell cycle defects, this could open opportunities for combination treatments with chemotherapies [317].

### Targeting SRPK, CLK, and DYRK kinase families in developmental disorders

As discussed in this review, SRPK, CLK, DYRK, and sub‐family HIPK have emerging roles in developmental disorders, particularly neurological indications, suggesting that they may be valuable therapeutic targets in this arena. With several neuropathologies linked to SRPK/CLK/DYRK kinase dysregulation, the applicability of inhibition strategies will be determined by the impact of gene disruption on specific kinases and/or downstream pathways. Specific kinases could be targeted to treat particular conditions, for example by reducing DYRK1A activity by chemical inhibition or targeted protein degradation (e.g. using proteolysis targeting chimeras, PROTACs). Inhibiting DYRK1A may reduce the risk of developing neurological dysfunction and neurodegeneration in Trisomy 21/Down's syndrome individuals with elevated DYRK1A levels. However, tissue‐specific and isoform‐selective DYRK1A inhibitors [318, 319] may be required, as DYRK family members have other key functions. For example, DYRK2 has pleotropic roles in cancer [272, 291] and can act as an apoptotic kinase [135], whilst DYRK1A/1B/3 are protective against apoptosis [320, 321] and important during interphase of the cell cycle [322]. In contrast, in other intellectual disabilities, such as DYRK1A‐related haploinsufficiency syndrome with *DYRK1A/1B* mutations and/or deletions, downstream signalling is likely impaired in ways that are not yet well understood [265, 266, 267, 270]. However, there are indications that it may be beneficial to restore activity to correct for imbalanced neuronal signalling and reduced GABAergic neurotransmission [268]. Therefore, strategies to stabilise overexpressed or amplified DYRK1A signalling (discussed earlier, e.g. *via* Notch, CyclinD1, p27(Kip1), REST [127, 210, 218, 220, 274, 275]) and/or reactivate downstream pathways will be necessary to manage or reverse adverse neurological symptoms caused by mutations in DYRK kinases.

In the case of the SRPK, the situation is similarly complex. As discussed earlier, *SRPK2* deletions and *SRPK3* point mutations have been identified in intellectual disability patients [93], which are predicted to disrupt SRPK kinase activity and downstream signalling. Indeed, variants in the SRPK substrate RNF12 disrupt E3 ubiquitin ligase activity and cause Tonne‐Kalscheuer syndrome intellectual disability [257], consistent with the notion that impaired SRPK signalling causes intellectual disability in patients. Therefore, restoring RNF12 substrate degradation using targeted protein degradation may be beneficial to restore balanced SRPK‐RNF12 signalling during neuronal development. However, there is also evidence of *SRPK3* [93] and *RNF12* [263] gene amplifications in intellectual disability, suggesting that elevated SRPK signalling may also lead to neurodevelopmental phenotypes. Therefore, to target SRPK, DYRK, and related kinases for treatment of a given neurological condition, the impact of human disease mutations on kinase activity, function, and downstream signalling must first be conclusively established.

### Summary

Further exploration is required to unpick the genetic phenotypes and novel functions of SRPK/CLK/DYRK families, and particularly how dysregulated SRPK and DYRK signalling leads to intellectual disabilities. For example, the regulation of ubiquitin‐mediated signalling and target degradation is an emerging function of SRPK, and as catalytic and adaptor functions impacting ubiquitin system have also been identified for DYRK and HIPK, it is likely that these kinases control protein turnover during developmental processes. Further research can aim to elucidate at which stages of development and cellular differentiation these kinases function. For example, how and when does SRPK affect induction and regulation of transcriptional activity beyond that of splicing (which is a dominant role for CLK)? Can proteomic and transcriptional networks be developed and interrogated to identify common nodes dependent on SRPK and DYRK during development and neural differentiation?

Genetic studies in model organisms are invaluable for characterising phenotypes associated with disrupted kinase function. With limited functional redundancy in lower model organisms, deciphering the developmental functions of the SRPK/CLK/DYRK kinase families has been possible. However, this needs to be extended to elucidate isoform‐specific functions in humans. One recent study highlights an interesting approach to tackling this problem, in which both some common functions and some non‐redundant functions of the four human HIPK isoforms were identified using a *Drosophila* model [256]. Moreover, the use of pluripotent stem cells is pivotal for understanding the role of kinase isoforms during differentiation and development. Using controlled stem cell models of differentiation, careful genetic and biochemical studies can be performed, generating multiple isoform knockouts and identifying unique and overlapping interactomes and phospho‐proteomes during stages of lineage‐specific differentiation. Furthermore, stem cell models are crucial for characterising the mechanistic impact and function of rare and novel patient mutations in specific kinases. These functions can inform further phenotypic studies in model organisms that may reflect and explain aspects of patient disorders. Alongside biological experimentation, it will be important to continue to develop appropriate chemical tools, as discussed above, to aid in the *in vivo* determination of kinase functions and the functional consequences of isoform‐specific or broader kinase family inhibition. Current tool compounds have been developed largely as a result of CMGC kinase involvement in varied disease indications, cellular contexts, and pathways not discussed in this review.

In all cases, a critical question concerns the timing of therapeutic interventions due to their strong associations with developmental phenotypes. For example, is it possible to treat symptoms in adults or only during early foetal development? Inhibition of several SRPK/CLK/DYRK kinases would likely cause damage to the developing embryo, where gene dosage balance is critical in development, especially in the nervous system. Furthermore, since overlapping substrates, interactors, and substrate motifs have been identified for SRPK/CLK/DYRK kinase families, further deconvolution is required for determining appropriate contexts for therapeutic intervention and where specific tools may need to be developed to delineate and target isoform‐specific effects. Nevertheless, the current data suggest that the SRPK/CLK/DYRK kinase families may represent important therapeutic targets in a variety of developmental disorders.

## Outlook

Protein phosphorylation dynamically regulates all aspects of cellular functions, from transcriptional activation to nutrient signalling, protein localisation, and cell cycle progression. Within the CMGC protein kinase superfamily, the SRPK/CLK/DYRK kinase families remain relatively poorly studied. However, there are emerging roles for these kinases in developmental signalling, stem cell regulation, and particularly in co‐ordinating neurological development and functioning. Disruption of these signalling pathways can underpin neurodevelopmental disorders such as intellectual disability and other neuropathologies, including neurodegeneration. We therefore consider neuronal cells as one of the key cell types in which SRPK and related kinases perform critical functions.

To unpick the mechanistic roles of specific kinases in the context of patients with intellectual disabilities and neurological disorders, models of neuronal differentiation and functioning will be important. Thus, pluripotent stem cells represent invaluable tools to delineate developmental mechanisms of interest through biochemical understanding of SRPK/CLK/DYRK kinase function during controlled neural and organoid differentiation, which can then inform approaches to model organism validation. In the future it will be interesting to unravel how these kinases finely balance the development of the nervous system, and how this is disrupted in neurological disorders, firstly for basic understanding and then to elaborate therapeutic strategies to improve the lives of patients.
